# Interaction effects of environmental factors with white blood cell profiles on mycobacterial pulmonary diseases: a case-control study

**DOI:** 10.1186/s12879-025-12375-3

**Published:** 2025-12-23

**Authors:** Xuan Ngoc Tran, Kuan-Jen Bai, Chi-Won Suk, Yuan-Chien Lin, Kian Fan Chung, Hsiao-Chi Chuang

**Affiliations:** 1 International Ph.D. Program in Medicine, College of Medicine, Taipei Medical University, Taipei, Taiwan; 2 Division of Pulmonary Medicine, Department of Internal Medicine, Wan Fang Hospital, Taipei Medical University, Taipei, Taiwan; 3 School of Respiratory Therapy, College of Medicine, Taipei Medical University, Taipei, Taiwan; 4 Department of Civil Engineering, National Central University, Taoyuan City, Taiwan; 5 National Heart and Lung Institute, Imperial College London, London, UK; 6 Division of Pulmonary Medicine, Department of Internal Medicine, Shuang Ho Hospital, Taipei Medical University, New Taipei City, Taiwan; 7 Cell Physiology and Molecular Image Research Center, Wan Fang Hospital, Taipei Medical University, Taipei, Taiwan; 8 Graduate Institute of Medical Sciences, College of Medicine, Taipei Medical University, Taipei, Taiwan; 9 Inhalation Toxicology Research Lab (ITRL), School of Respiratory Therapy, College of Medicine, Taipei Medical University, 250 Wuxing Street, Taipei, 11031 Taiwan

**Keywords:** DS-TB, MDR-TB, NTM, Meteorological factors, Immunity response

## Abstract

**Background:**

Mycobacterial pulmonary diseases remain a significant global health concern. This study investigates the interaction effects of relative humidity (RH), temperature, and fine particular matter (PM_2.5_) with immune profiles, reflected by white blood cell (WBC) counts, on pulmonary drug-susceptible tuberculosis (DS-TB), multidrug-resistant TB (MDR-TB), and non-tuberculous mycobacteria (NTM) disease.

**Methods:**

This case-control study of 1,398 participants, including 409 cases (203 DS-TB, 151 MDR-TB, and 55 NTM) and 989 controls, assessed individual exposure to RH, temperature, and PM_2.5_, in mean and difference over 1-month interval, using radial basis function interpolation. Logistic regression models evaluated the associations between environmental exposures and these mycobacterial diseases while also analyzing their interaction effects with WBCs. Generalized additive models with penalized splines were used to explore potential non-linear associations.

**Results:**

Higher mean RH was associated with a 0.92-fold decreased OR for DS-TB (95% CI: 0.88, 0.96), while higher temperature daily difference and PM_2.5_ were associated with higher ORs across all mycobacterial disease groups. Non-linear models illustrated U-shape associations for those environmental factors. Additionally, elevated neutrophil levels attenuated the impact of temperature daily difference on NTM disease, while higher lymphocyte levels amplified temperature daily difference-related effects for DS-TB and MDR-TB.

**Conclusion:**

This study highlights the role of WBC profiles in modifying the relationship between short-term temperature exposure and mycobacterial pulmonary disease, underscoring the interplay between environmental triggers and immune profiles in disease pathogenesis. Understanding these associations may enhance strategies for TB prevention and early detection.

**Clinical trial number:**

Not applicable.

**Supplementary Information:**

The online version contains supplementary material available at 10.1186/s12879-025-12375-3.

## Introduction

Tuberculosis (TB), caused by *Mycobacterium tuberculosis* (MTB), is an ancient infectious disease that remains a major global health concern [1]. Multidrug-resistant TB (MDR-TB), defined as MTB that is resistant to at least rifampicin and isoniazid, presents a significant health challenge, with only one-third of patients receiving appropriate treatment [2]. Additionally, nontuberculous mycobacterial (NTM) pulmonary diseases, caused by diverse environmental mycobacteria such as *M. avium* and *M. intracellulare*, have gained increasing attention in recent decades due to their rising incidence, complex treatment challenges, and growing contribution to morbidity and healthcare burden [3]. As the majority of NTMs are environmental organisms, the impact of global climate change on their ecology and transmission is an emerging concern [4]. This highlights the urgent need for further research into these mycobacterial pulmonary diseases. While the bacterium itself is crucial in disease pathogenesis, environmental factors and host immune responses play significant roles in influencing disease susceptibility [5–11]. Environmental factors such as relative humidity (RH), temperature, and fine particulate matter (PM_2.5_) have been shown to influence the transmission of MTB and risk of developing TB and NTM [5–9]. The immune system is essential, as imbalances in white blood cells (WBCs) are associated with poor TB outcomes [10, 11]. These conditions share partially overlapping environmental and host immune determinants, while differing in microbial species and transmission dynamics [4–9, 12, 13]. Examining both diseases in parallel allows for direct comparison of shared and distinct exposure-response patterns.

Previous studies have primarily examined the independent impacts of environmental exposures and host immune responses on mycobacterial diseases [5–11, 14, 15]. In real-world settings, TB development results from a complex interplay between environmental stressors and host immune responses, rather than the influence of either factor in isolation [16]. The World Health Organization has established a goal to eliminate the worldwide TB epidemic by 2035, targeting a 90% decrease in TB incidence rates and a 95% decline in TB-related deaths [17]. Among the multifaceted efforts to eliminate tuberculosis, understanding the complex relationship between environmental factors, host immunity, and the disease is essential for improving prevention and control strategies. However, it remains unclear whether susceptibility to environmental exposures varies by an individual’s immune profile. While TB and NTM typically have a long incubation period [18, 19], we hypothesize that short-term environmental stressors may act as proximal triggers that, in combination with a susceptible immune response, are associated with the onset and clinical detection of active mycobacterial disease. Assessing environmental exposures in the 1-month period prior to diagnosis may thus capture proximate environmental conditions influencing disease occurrence or detection, rather than the initial risk of infection. The objective of this study is to examine the interaction effects of RH, temperature, and PM_2.5_ with immune profiles, reflected by WBC counts, on pulmonary DS-TB, MDR-TB, and NTM. Findings may help identify immune profiles more vulnerable to environmental stressors and inform more targeted TB control strategies.

## Materials and methods

### Study design and population

**Fig. 1:**
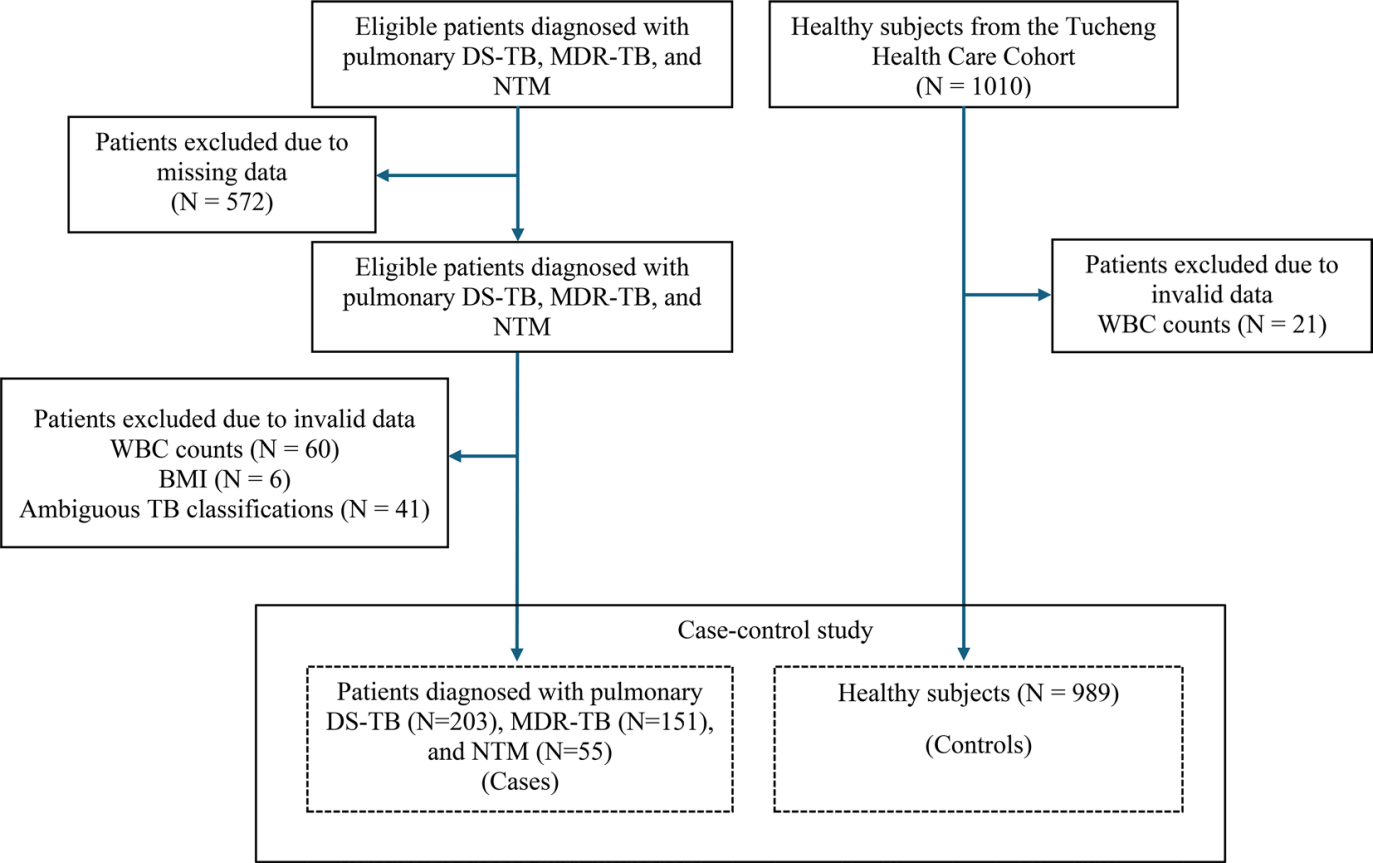
Flow chart of study subjects

This case-control study involved a total of 1,398 participants, consisting of 409 cases diagnosed with pulmonary DS-TB (TB without resistance to rifampicin and isoniazid), MDR-TB, and NTM, along with 989 healthy controls (Fig. 1). Both cases and controls were drawn from the same metropolitan area (New Taipei City, Taiwan), ensuring that all participants experienced comparable environmental conditions and healthcare accessibility. TB and NTM patients aged between 19 and 90 years were recruited from the chest clinic of the Northern Taiwan TB referral center, located in New Taipei City, Taiwan [20]. These patients had a newly confirmed diagnosis of pulmonary DS-TB, MDR-TB or NTM at the time of enrollment, during the period from 1 April 2014 to 30 November 2022. Control subjects aged 40–90 years were recruited from the Tucheng Health Care Cohort, a community-based health screening program conducted between 2018 and 2021 in the Tucheng District of New Taipei City, Taiwan [21]. It was established to investigate environmental factors and chronic diseases through standardized questionnaires (see Supplementary document), physical examinations, blood testing, and pulmonary assessments [21]. The cohort provides a reliable community-based source of control participants without evidence of active infection. The exclusion criteria for this study included individuals undergoing anticancer therapy or testing positive for human immunodeficiency virus.

### Data collection

Medical data, including age, sex, body mass index (BMI), smoking status, WBC profiles and diagnosis were systematically documented for all participants. All patients had newly confirmed diagnoses and were enrolled before the initiation of anti-mycobacterial treatment (standard first-line therapy for DS-TB, second-line therapy for MDR-TB, and species-specific antibiotic regimens for NTM), ensuring that blood samples reflected pre-treatment immune profiles. No additional procedures were conducted for the study. All WBC measurements, including neutrophil, eosinophil, and lymphocyte counts, were performed as part of routine diagnostic work-up at the time of initial evaluation for differentiation of pulmonary mycobacterial diseases. Informed consent was waited due to the retrospective nature of the study and use of anonymized data.

### Diagnosis of pulmonary TB, MDR-TB, and NTM

Study subjects were divided into individuals diagnosed as pulmonary DS-TB, MDR-TB, and NTM. The diagnoses of pulmonary DS-TB, MDR-TB, and NTM were confirmed through the identification of mycobacteria through culture-positive sputum samples [22, 23]. Pulmonary DS-TB and MDR-TB were differentiated from NTM by their colony morphology, bacterial growth patterns, immunochromatographic test, and polymerase chain reaction test. MTB isolates underwent drug susceptibility testing via the BACTEC MGIT (Mycobacteria Growth Indicator Tube) method to evaluate resistance to anti-tuberculosis drugs [24].

### Individual-level exposure to ambient relative humidity, temperature and PM_2.5_

Individual-level exposure to environmental factors was estimated for each individual based on residential addresses recorded at the time of recruitment. Hourly RH and temperature information were obtained from the Taiwan Central Weather Bureau, while hourly PM_2.5_ concentrations were collected from monitoring stations managed by the Taiwan Environmental Protection Administration. PM_2.5_ concentration measurements were validated against data obtained from air quality monitoring instruments, showing strong agreement (R² = 0.93, *p* < 0.001) [25]. The collected hourly data underwent reorganization and cleaning, followed by spatial estimation for each participant. To align with the temporal scale of participant data, hourly measurements were averaged into daily mean values. The radial basis function (RBF) interpolation method was used to estimate the spatial distribution of RH, temperature, and PM_2.5_ exposure across Taiwan, utilizing the coordinates of each data point as described in previous research [26].The RBF approach is recognized as one of the most precise spatial interpolation techniques for environmental applications, capable of generating smooth and continuous concentration surfaces from unstructured monitoring data. It is a mesh-free method that accurately represents spatial variability without requiring a regular grid, producing high-quality estimates across complex terrains [27]. This study aimed to assess the impact of short-term exposure; however, given that tuberculosis has an incubation period ranging from one month to several years, a lag period of 30 days for incubation period, which refer to the time delay between environmental exposure and TB diagnosis was applied [28]. To assess exposure effects, both daily mean values and differences of these exposures were calculated over 30-day intervals. The 1-month mean value was derived as the average of daily mean values from 29th day before the case up to the case day. The daily difference was determined by comparing the mean value of the current day with that of the previous day. The 1-month daily difference was then determined as the average of daily difference values over this 30-day period. The detailed methods for calculating these means and daily differences in environmental variables have been described in our prior report [29].

### Data analysis

**Fig. 2:**
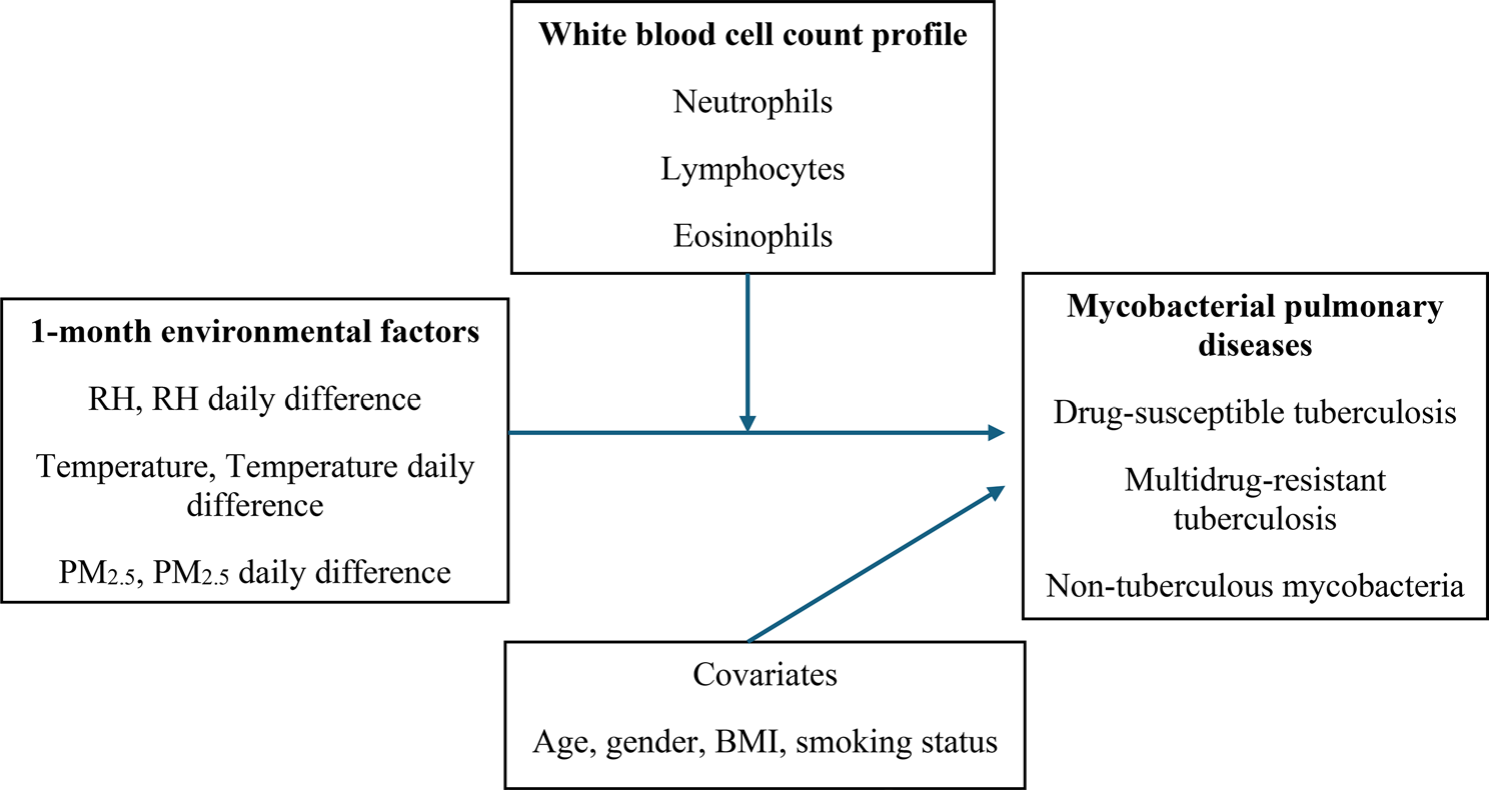
Directed Acyclic Graph illustrating associations among environmental exposures, white blood cell profiles, and pulmonary mycobacterial diseases

Continuous variables following a normal distribution were represented as means with standard deviations (SD), while categorical variables were reported as counts and percentages. To reduce the influence of outliers, extreme values exceeding the 99th percentile were modified using the Winsorization technique [30]. Comparisons of continuous variables were performed using one-way ANOVA for normally distributed data. For categorical variables, the Chi-squared test was used. The associations of environmental exposures with mycobacterial pulmonary diseases were first analyzed using logistic regression. We further examined potential non-linear associations by constructing dose-response curves illustrating changes in the odds ratios (ORs) of mycobacterial diseases across varying levels of environmental exposures. These relationships were modeled using generalized additive models (GAMs) with penalized splines. The maximum spline flexibility was limited by specifying four basis functions (k = 4), as supported by previous literature and model parsimony considerations [31], while the optimal degree of smoothness was automatically determined using the generalized cross-validation (GCV) criterion implemented in the GAM framework. Interaction terms between environmental exposures and WBC profiles were added to the logistic regression models in addition to the main effects to explore which immune profiles are more vulnerable to environmental stressors. Covariates, including age, sex, BMI, and smoking status, were adjusted in all models. All statistical computations were performed using R software with the MGCV package (version 4.2.2). To account for multiple testing across environmental variables and WBC parameters, p-values were adjusted using the Benjamini-Hochberg false discovery rate (BH-FDR) correction. Results with FDR-adjusted p-values < 0.05 were considered statistically significant. A sensitivity analysis was conducted for the NTM-control group using propensity-score matching to confirm the robustness of the findings, given the significant sample size imbalance between the NTM and control groups. Nearest-neighbor 1:1 matching without replacement was applied, yielding 55 NTM cases and 55 matched controls with balanced covariates (standardized mean differences < 0.15 for all variables). Logistic regression models were then refitted on the matched dataset to re-evaluate associations between environmental predictors and NTM, with p-values again adjusted by the BH-FDR correction. A directed acyclic graph was developed to depict the association among environmental exposures, WBC profiles, and pulmonary mycobacterial diseases (Fig. 2).

## Results

### Characteristics of study subjects

Table 1 (see the end of the manuscript) shows the basic characteristics of 1,398 participants, including 409 cases (203 DS-TB, 151 MDR-TB, and 55 NTM) and 989 controls. Pulmonary DS-TB, MDR-TB, and NTM accounted for 49.6%, 36.9%, and 13.5% of the case group, respectively (Fig. 2; Table 1). The mean age was 64.7 ± 21.8 years in the DS-TB group and 56.4 ± 20.5 years in the MDR-TB group. Among all groups, males accounted for 58.2–65.0% of cases and 29.5% of controls. Mean BMI ranged from 21.1 to 21.5 kg/m² in the case groups and 24.4 kg/m² in controls. Current smokers represented 29.1–41.7% of cases and 67.6% of controls. Mean neutrophil counts ranged from 3.4 ± 1.4 to 6.1 ± 4.2 × 10³ cells/µL, eosinophil counts from 0.1 ± 0.1 to 0.2 ± 0.4 × 10³ cells/µL, and lymphocyte counts from 1.3 ± 1.0 to 2.0 ± 0.6 × 10³ cells/µL across all groups. The 1-month mean relative humidity (RH) ranged from 74.6 ± 4.4% to 76.3 ± 4.8%, and RH daily difference ranged from − 0.1 ± 4.3% to 1.0 ± 4.2% across all groups. Mean daily temperature difference was largest in the DS-TB group (1.1 ± 3.3 °C). Mean PM_2.5_ concentration and PM_2.5_ daily difference were highest in the NTM (17.3 ± 7.2 µg/m³) and DS-TB (1.7 ± 5.9 µg/m³) groups, respectively.

### Association of RH, temperature and PM_2.5_ with pulmonary DS-TB, MDR-TB, and NTM

Table 2 shows the associations between 1-month mean values and daily differences in RH, temperature and PM_2.5_ and pulmonary mycobacterial diseases, including DS-TB, MDR-TB, and NTM. A 1% increase in mean RH was associated with a 0.92-fold decrease in OR for DS-TB (95% CI: 0.87–0.96). A 1 °C increase in temperature daily difference corresponded to an increased OR of 1.27-fold for DS-TB (95% CI: 1.18–1.36), 1.16-fold for MDR-TB (95% CI: 1.07–1.26), and 1.20-fold for NTM (95% CI: 1.07–1.35). Similarly, higher mean PM_2.5_ concentrations were associated with increased OR of disease, with OR of 1.14 (95% CI: 1.10–1.19) for DS-TB and 1.20 (95% CI: 1.12–1.28) for NTM. PM_2.5_ daily differences were also positively associated with pulmonary mycobacterial diseases, though only the result for TB was significant (OR: 1.11; 95% CI: 1.06–1.16).

**Table 1:** Basic characteristics of the study subjects

Characteristics	DS-TB (*N* = 203)	MDR-TB (*N* = 151)	NTM (*N* = 55)	Control (*N* = 989)	*p*-value
Age (years), mean ± SD	64.7 ± 21.8	56.4 ± 20.5	66.9 ± 19.9	63.4 ± 8.1	0.07
Gender, n (%) Female Male	71 (35.0) 132 (65.0)	56 (37.1) 95 (62.9)	23 (41.8) 32 (58.2)	697 (70.5) 92 (29.5)	< 0.05
Body mass index (BMI, kg/m^2^), mean ± SD	21.1 ± 3.6	21.5 ± 3.8	21.4 ± 3.9	24.4 ± 3.5	< 0.05
Smoking status, n (%) Non-smoker Smoker	131 (64.5) 72 (35.5)	88 (58.3) 63 (41.7)	39 (70.9) 16 (29.1)	320 (32.4) 669 (67.6)	< 0.05
White blood cells (10³ cells/µL), mean ± SD Neutrophils Lymphocytes Eosinophils	6.1 ± 4.2 1.3 ± 1.0 0.2 ± 0.4	4.5 ± 2.4 1.5 ± 0.9 0.2 ± 0.4	5.7 ± 3.4 1.5 ± 0.9 0.2 ± 0.3	3.4 ± 1.4 2.0 ± 0.6 0.1 ± 0.1	< 0.05
Environmental exposure, mean ± SD (min-max) Humidity (%) Humidity difference (%) Temperature (°C) Temperature difference (°C) PM_2.5_ (µg/m^3^) PM_2.5_ difference (µg/m^3^)	74.6 ± 4.4 (66.7, 84.8) 0.6 ± 4.7 (-9.0, 9.5) 23.2 ± 4.8 (15.1, 30.7) 1.1 ± 3.3 (-4.9, 7.6) 16.1 ± 6.0 (7.1, 36.0) 1.7 ± 5.9 (-7.2, 19.4)	76.3 ± 4.8 (66.7, 86.9) 0.8 ± 4.3 (-8.9, 9.5) 24.0 ± 4.7 (15.1, 30.7) 0.3 ± 3.0 (-4.9, 7.6) 14.2 ± 5.4 (7.1, 32.0) 1.2 ± 5.6 (-7.2, 19.2)	75.3 ± 4.3 (66.7, 83.9) 1.0 ± 4.2 (-6.7, 9.5) 22.3 ± 4.7 (15.5, 30.7) 0.8 ± 3.3 (-4.9, 7.6) 17.3 ± 7.2 (7.1, 36.1) 0.5 ± 5.1 (-7.2, 13.8)	76.0 ± 3.6 (67.7, 86.9) -0.1 ± 4.3 (-10.0, 7.6) 23.1 ± 4.8 (15.1, 30.7) -0.5 ± 2.4 (-4.9, 4.0) 13.6 ± 3.7 (8.1, 30.7) 0.3 ± 3.0 (-7.2, 7.6)	< 0.05 < 0.05 0.072 < 0.05 < 0.05 < 0.05

Abbreviations: SD, standard deviation; DS-TB, Drug-susceptible tuberculosis; MDR-TB, multidrug-resistant tuberculosis; NTM, non-tuberculous mycobacteria; PM_2.5_, particulate matter of *<* 2.5 μm in aerodynamic diameter

**Table 2:** Association of environmental factors and pulmonary DS-TB, MDR-TB and NTM

	DS-TB vs. Control OR (95%CI)	MDR-TB vs. Control OR (95%CI)	NTM vs. Control OR (95%CI)
Humidity (%) Humidity difference (%) Temperature (°C) Temperature difference (°C) PM_2.5_ (µg/m^3^) PM_2.5_ difference (µg/m^3^)	**0.92 (0.87**, 0.96) * 1.04 (1.00, 1.08) 1.00 (0.96, 1.04) **1.27 (1.18**, 1.36) * **1.14 (1.10**, 1.19) * **1.11 (1.06**, 1.16) *	1.05 (0.99, 1.10) 1.04 (0.99, 1.09) 1.03 (0.99, 1.08) **1.16 (1.07**, 1.26) * 1.04 (1.01, 1.09) 1.06 (1.01, 1.12)	0.99 (091, 1.08) 1.08 (0.99, 1.16) 0.93 (0.87, 1.00) **1.20 (1.07**, 1.35) * **1.20 (1.12**, 1.28) * 1.03 (0.94, 1.13)

Abbreviations: PM_2.5_, particulate matter of *<* 2.5 μm in aerodynamic diameter; DS-TB, drug-susceptible tuberculosis; MDR-TB, multidrug-resistant tuberculosis; NTM, non-tuberculous mycobacteria; OR, Odds Ratio; CI, confidence interval. Age, gender, BMI, and smoking status were adjusted for in the models. Bolded results are considered significant; * denotes adjusted *p* < 0.05 after Benjamini-Hochberg false discovery rate correction

### Non-linear effects of RH, temperature and PM_2.5_ with pulmonary DS-TB, MDR-TB, and NTM

**Fig. 3:**
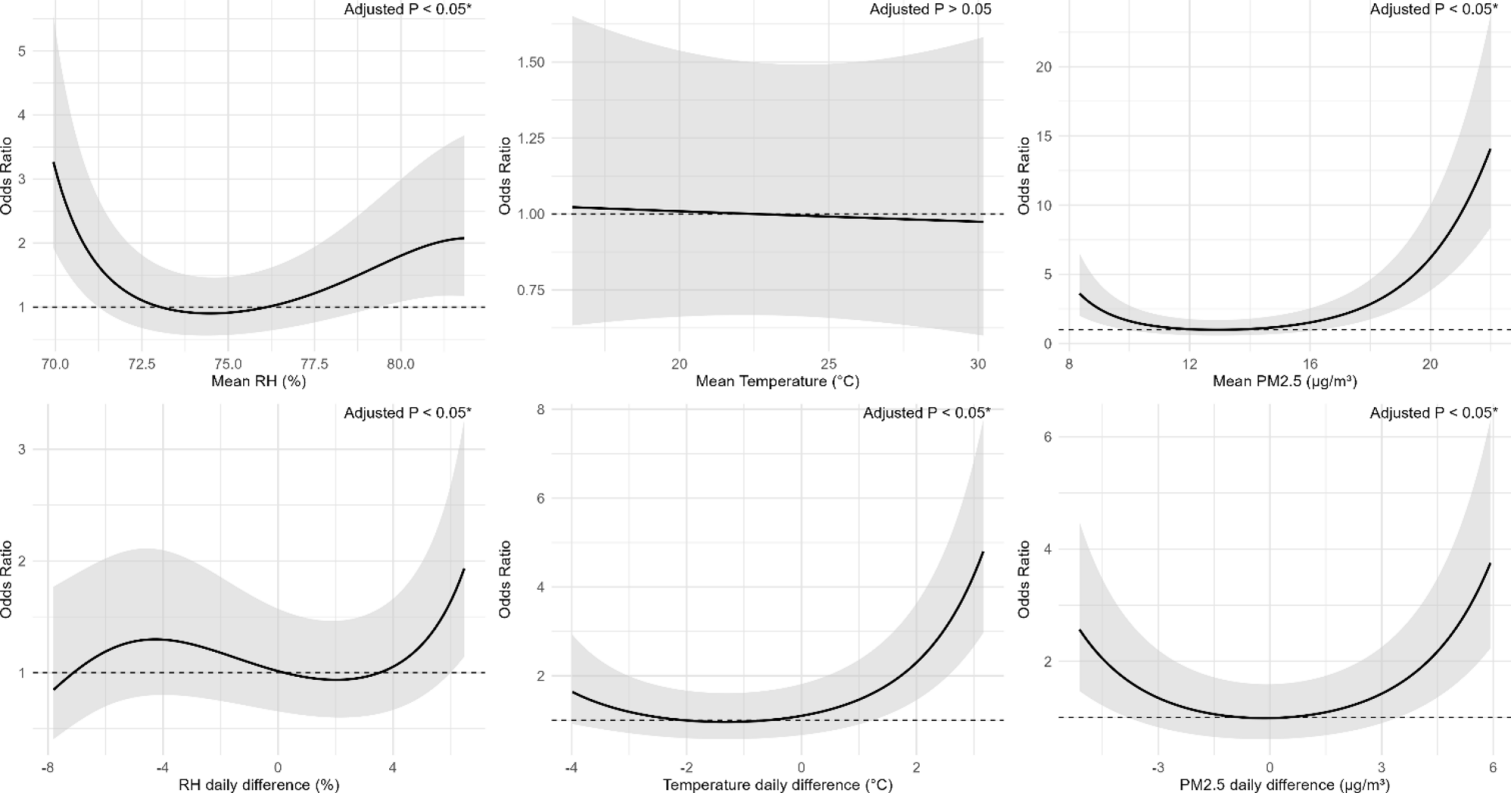
Exposure-response relationship between the relative humidity (RH), temperature, PM_2.5_ and pulmonary DS-TB. Panels show the associations for (**a**) mean RH (%), (**b**) mean temperature (°C), (**c**) mean PM_2.5_ (µg/m³), (**d**) RH daily difference (%), (**e**) temperature daily difference (°C), and (f) PM_2.5_ daily difference (µg/m³). The solid line represents the odds ratio (OR) for DS-TB. The shaded area shows the 95% CI, reflecting the precision of the OR estimate. Covariates adjusted for in the models were age, gender, BMI, and smoking status. Asterisks (*) mark statistically significant associations where adjusted *p* < 0.05 after Benjamini–Hochberg false discovery rate (BH-FDR) correction. The dashed line across the OR of 1.0 serves as a reference

**Fig. 4:**
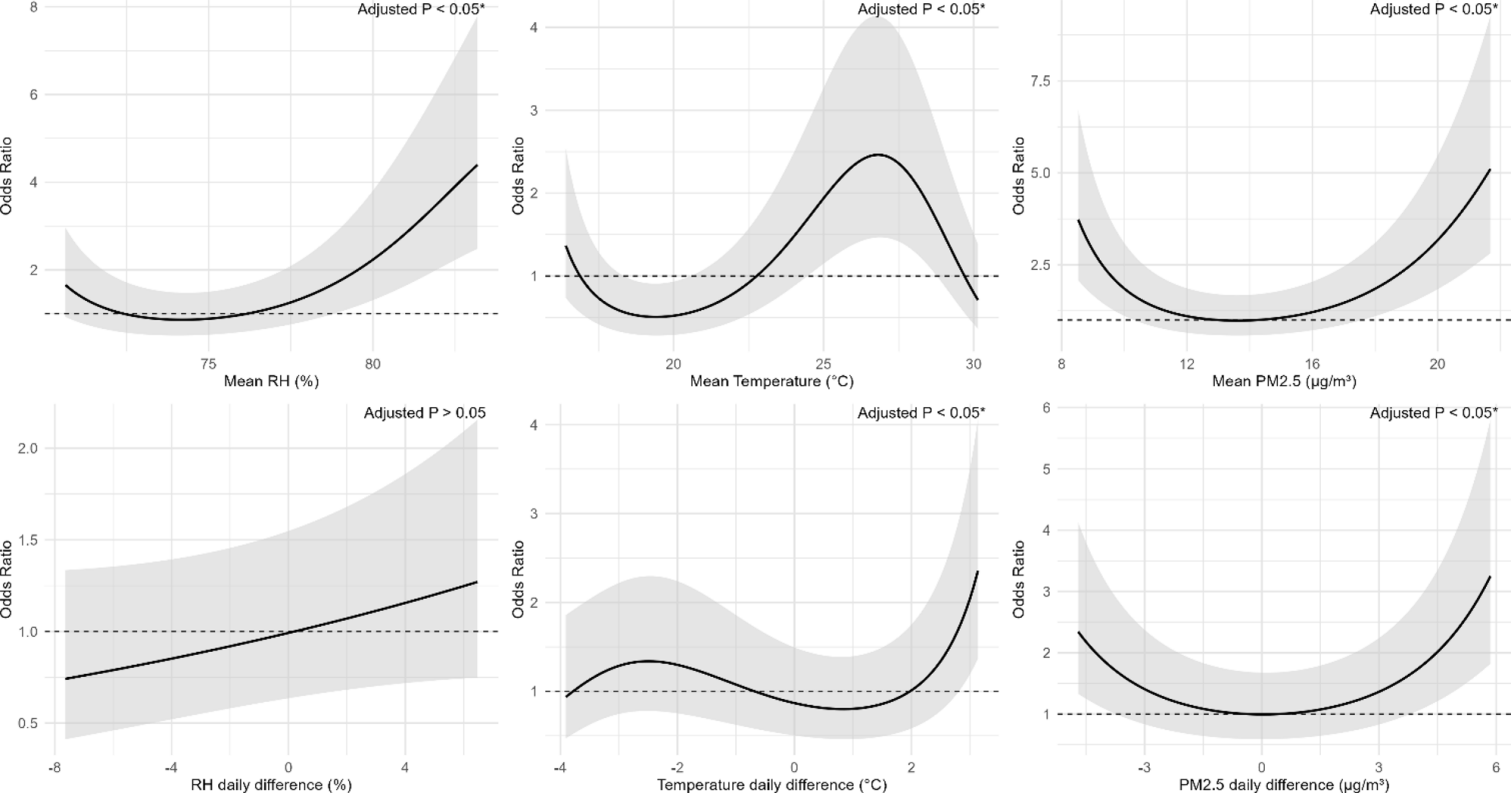
Exposure–response relationship between the relative humidity (RH), temperature, PM_2.5_ and pulmonary MDR-TB. Panels show the associations for (**a**) mean RH (%), (**b**) mean temperature (°C), (**c**) mean PM_2.5_ (µg/m³), (d) RH daily difference (%), (**e**) temperature daily difference (°C), and (**f**) PM_2.5_ daily difference (µg/m³). The solid line represents the odds ratio (OR) for MDR-TB. The shaded area shows the 95% CI, reflecting the precision of the OR estimate. Covariates adjusted for in the models were age, gender, BMI, and smoking status. Asterisks (*) mark statistically significant associations where adjusted *p* < 0.05 after BH-FDR correction. The dashed line across the OR of 1.0 serves as a reference

**Fig. 5:**
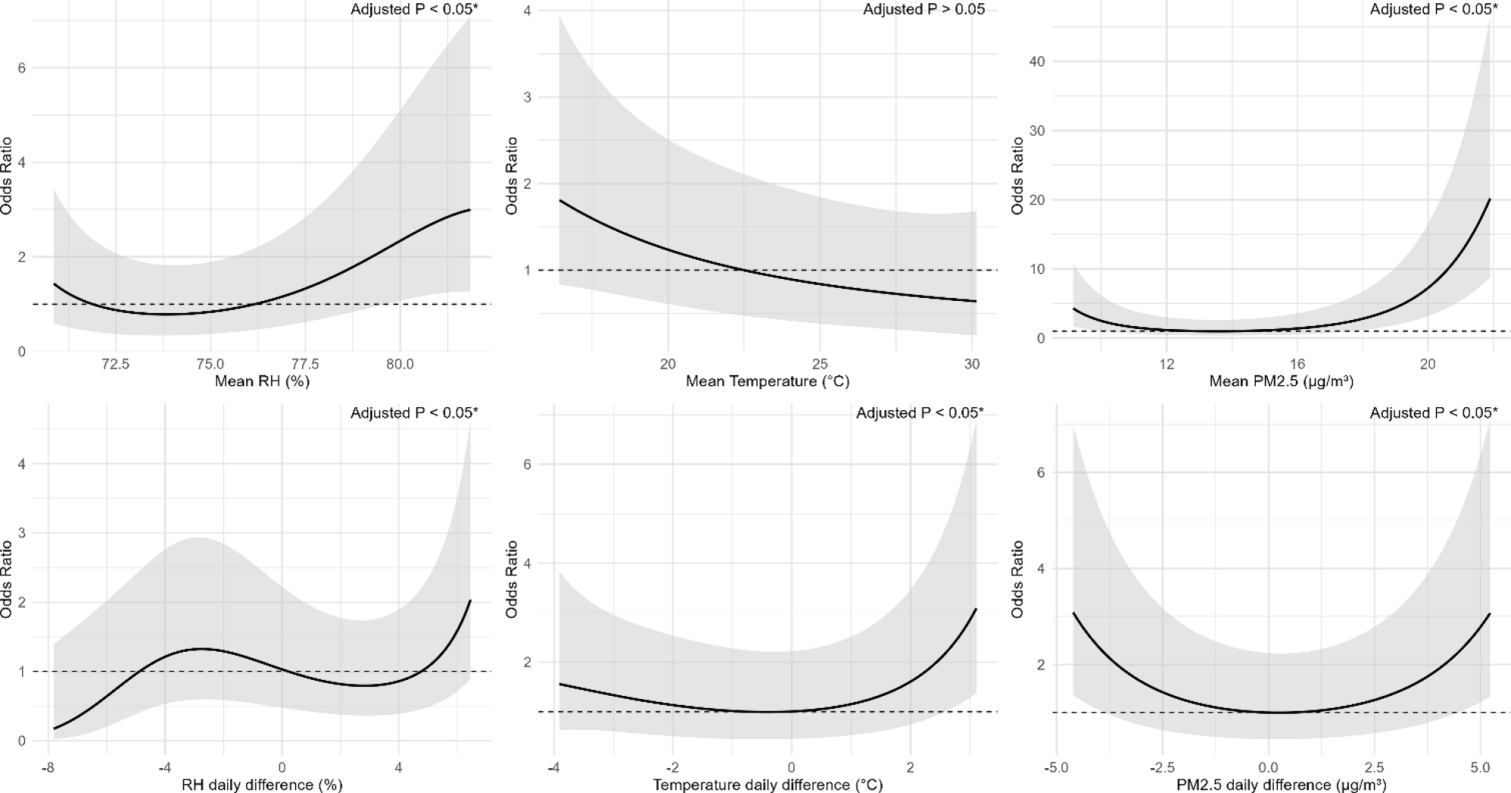
Exposure–response relationship between the relative humidity (RH), temperature, PM_2.5_ and pulmonary NTM. Panels show the associations for (**a**) mean RH (%), (**b**) mean temperature (°C), (**c**) mean PM_2.5_ (µg/m³), (d) RH daily difference (%), (**e**) temperature daily difference (°C), and (**f**) PM_2.5_ daily difference (µg/m³). The solid line represents the odds ratio (OR) for NTM. The shaded area shows the 95% CI, reflecting the precision of the OR estimate. Covariates adjusted for in the models were age, gender, BMI, and smoking status. Asterisks (*) mark statistically significant associations where adjusted *p* < 0.05 after BH-FDR correction. The dashed line across the OR of 1.0 serves as a reference

Figures 3 and 4, and 5 (see the end of the manuscript) illustrate the non-linear associations between RH, temperature and PM_2.5_ with pulmonary DS-TB, MDR-TB, and NTM. Across all three diseases, RH demonstrated U-shaped associations, where both low and high humidity levels were associated with increased ORs. RH daily difference showed an increased risk at higher daily difference, especially beyond 4%. Mean temperature showed a flat or slightly decreasing trend, with the lowest OR near 23 °C, but this association was not statistically significant. Temperature daily difference exhibited a U-shaped relationship, with the lowest risk observed around 0 °C difference. Both large decreases (below − 3 °C) and increases (above 3 °C) in daily temperature difference were associated with elevated ORs. For all three diseases, higher PM_2.5_ mean and daily difference also displayed U-shaped curves, with the lowest risk around 13 µg/m³ for mean PM_2.5_ and near 0 µg/m³ for PM_2.5_ daily difference.

### Association of WBC profiles with pulmonary DS-TB, MDR-TB and NTM

Table 3 illustrates the associations of WBC counts with mycobacterial diseases, reflecting immune response profiles at the time of diagnosis. A 10³ cells/µL increase in neutrophil count was associated with increased ORs across all case groups, with the most pronounced effect observed in DS-TB (OR:1.65, 95% CI: 1.47–1.86). Additionally, a 10³ cells/µL increase in eosinophil count corresponded to a 4.47-fold higher OR for MDR-TB (95% CI: 1.93–12.10). In contrast, a 10³ cells/µL increase in lymphocyte count inversely associated with all case groups, with OR of 0.29 (95% CI: 0.20–0.39) for DS-TB, OR of 0.34 (95% CI: 0.23–0.48) for MDR-TB, and OR of 0.34 (0.20–0.56) for NTM.

**Table 3:** Association of white blood cells with pulmonary DS-TB, MDR-TB, and NTM

	DS-TB vs. Control OR (95%CI)	MDR-TB vs. Control OR (95%CI)	NTM vs. Control OR (95%CI)
Neutrophils (10³ cells/µL) Lymphocytes (10³ cells/µL) Eosinophils (10³ cells/µL)	**1.65 (1.47**, 1.86) * **0.29 (0.20**, 0.39) * 1.99 (0.91, 4.37)	**1.24 (1.11**, 1.40) * **0.34 (0.23**, 0.48) * **4.47 (1.93**, 12.10) *	**1.46 (1.27**, 1.70) * **0.34 (0.20**, 0.56) * 2.50 (0.58, 11.70)

Abbreviations: DS-TB, drug-susceptible tuberculosis; MDR-TB, multidrug-resistant tuberculosis; NTM, non-tuberculous mycobacteria; CI, confidence interval; OR, Odds Ratio. Age, gender, BMI, and smoking status were adjusted for in the models. Bolded results are considered significant; * denotes adjusted *p* < 0.05 after Benjamini–Hochberg false discovery rate correction

### Interaction effects of RH, temperature and PM_2.5_ with immune profiles on pulmonary DS-TB, MDR-TB, and NTM

Table 4 (see the end of the manuscript) demonstrates the interaction between environmental exposures and immune profiles on pulmonary mycobacterial diseases. A 1 °C increase in temperature daily difference decreased the OR of NTM by 14% (OR:0.86; 95% CI: 0.81–0.91) for each 10³ cells/µL increase in neutrophils, beyond their individual effects. In contrast, the interaction between temperature daily difference and lymphocytes raised the OR of DS-TB by 25% (OR: 1.25; 95% CI: 1.13–1.39), and MDR-TB by 17% (OR: 1.17; 95%CI: 1.03–1.34) compared with the absence of interaction. No significant interaction of eosinophils and environmental exposures on these infections were observed.

Sensitivity analysis for the NTM group showed similar results to the primary analysis, with all significant associations from the primary analysis retaining statistical significance. Although there were slight changes in the OR and 95% CIs after applying PSM, the overall patterns remained consistent (Table S1, S2, S3).

## Discussion

While MTB remains the primary pathogen driving TB, previous studies have demonstrated that environmental exposures can influence TB development, especially in the context of rapidly changing climate. In our study, we examined susceptibility to pulmonary DS-TB, MDR-TB, and NTM by incorporating environmental conditions and host immunity, specifically WBC profiles. The key novelty of this study lies in identifying that elevated neutrophil levels attenuated the effects of temperature daily difference on the diagnosis of NTM, whereas increased lymphocyte levels amplified this temperature daily difference -related associations with DS-TB and MDR-TB. This research highlights the crucial role of temperature daily difference with WBC profiles in pulmonary mycobacterial diseases.

Our linear models revealed that the odds of mycobacterial diseases decreased with higher 1-month mean RH, but the odds increased with greater temperature daily differences and PM_2.5_ metrics. A negative association between RH and TB has been reported in previous researches in China [14] and Vietnam [15]. One possible explanation is that lower humidity can induce airway constriction and dry out the mucosal lining [32], potentially exacerbating inflammation and impairing mucosal defense, thereby facilitating the progression from latent infection to active TB disease, whereas higher ambient humidity may help preserve mucosal integrity, which could lower the likelihood of disease manifestation [5]. Regarding temperature difference, our finding is consistent with a previous study, indicating that exposure to significant temperature drops between consecutive days and large diurnal temperature ranges were linked to an increased risk of TB manifestation [33]. There are several possible explanations for the consistent significant effects of temperature differences on pulmonary mycobacterial diseases. Sudden positive fluctuations in the temperature of inhaled air can trigger the release of inflammatory mediators from mast cells, potentially leading to inflammation of the airway tract [34]. Additionally, significant exposure to the diurnal temperature range may further increase heart rate, blood pressure, and oxygen uptake, which increase burden on both the lungs and heart [35], contributing to the onset or clinical detection of mycobacterial pulmonary diseases, including TB and NTM. In contrast, short-term exposure to large temperature drops can induce bronchoconstriction and airway obstruction [36] as well as epithelial injury in the respiratory tract [33, 34], leading to the potential exacerbation of pulmonary mycobacterial diseases. In terms of PM_2.5_, inhalable particulates contribute to active TB disease by damaging the tracheobronchial mucosa, impairing cytokine synthesis and secretion, and compromising systemic immune function, particularly anti-mycobacterial T cell immunity in the lungs, thereby promoting the progression to clinically detectable disease [7, 37].

Non-linear models, however, illustrated U-shape associations for 1-month mean RH, temperature differences and PM_2.5_ metrics. A U-shaped pattern of RH was also found for respiratory symptoms in prior studies, both low and high extremes in relative humidity are associated with increased respiratory symptoms [38]. Excessive humidity can impair the integrity of the airway epithelium, potentially enhancing viral infectivity or promoting fungal proliferation, thereby increasing susceptibility to respiratory conditions such as mycobacterial infections [39]. A U-shaped relationship between temperature daily difference and TB risk was also reported in a previous study [33], and the possible mechanisms underlying this association have been discussed above. Low PM_2.5_ may lead to insufficient immune stimulation [40], while high levels can damage the airway epithelium, induce oxidative stress, and impair immune defenses [37]. PM_2.5_ daily difference levels may further destabilize respiratory immunity by repeatedly stressing the mucosa, increasing vulnerability to infection [41]. These findings emphasize the need to evaluate both the mean levels and differences in environmental conditions when assessing these infections. They also suggest that associations may appear linear within specific exposure ranges but become non-linear as environmental levels exceed a physiological threshold.

Next, our study showed elevated neutrophil and eosinophil counts, and decreased lymphocyte levels being associated with TB and NTM, which may reflect distinct immune responses at the time of clinical presentation of mycobacterial infections. Neutrophils play a central role in the innate immune response against mycobacterial infections, serving as one of the first responders to infection sites [42]. Therefore, their increased counts in patients with TB and NTM likely reflect an active immune response to the presence of mycobacteria. Although eosinophils are primarily involved in allergic reactions and parasitic infections, emerging evidence suggests that they may participate in the granulocyte response in TB, potentially influencing disease progression and immune modulation [11]. In contrast, lymphocytes, especially CD4⁺ T cells, are essential for the adaptive immune response against MTB [43]. Lower lymphocyte counts observed in patients with TB and NTM align with the well-documented physiological stress response, where infection-induced inflammation leads to increased neutrophil release and relative lymphopenia [44]. This lymphocyte reduction may also reflect redistribution to infection sites or immune dysregulation during active disease [45]. Our findings highlight that elevated neutrophil and eosinophil counts, together with reduced lymphocyte levels, reflect characteristic immune response patterns during active mycobacterial infection at the time of diagnosis rather than pre-existing immune alterations. Although these immune alterations were observed in patients with confirmed TB or NTM, further investigations are needed to confirm these findings and clarify whether the observed immune profiles are specific to mycobacterial diseases or reflect broader inflammatory responses.

Finally, our findings suggest that WBC profiles may modify the relationship between short-term temperature daily difference exposure and mycobacterial disease detection, highlighting a potential interplay between environmental triggers and WBC responses in TB and NTM pathogenesis. Specifically, higher neutrophil levels attenuated the effects of temperature daily difference on the diagnosis of NTM, while increased lymphocyte counts amplified temperature daily difference-related associations in TB. The physiological mechanisms underlying the impact of temperature daily difference on NTM diminished among patients with higher neutrophil levels remain unclear; however, one possible explanation is that neutrophils are critical early responders to Mycobacterium exposure that phagocytose bacteria, release antimicrobial peptides, and form neutrophil extracellular traps, all of which contribute to enhance mucosal defense [42]. These protective functions may be particularly important under conditions of temperature daily difference-related stress, as elevated temperature fluctuations can impair mucosal barriers and dysregulate immune responses [33]. Individuals with more robust neutrophil activity may be better equipped to counteract these environmental insults, thereby reducing the impact of temperature on NTM. Regarding lymphocytes, the mechanism for the observation that the effects of temperature on TB were amplified among individuals with relatively higher lymphocyte levels is also not clear. This may be because elevated lymphocyte counts reflect a dysregulated or misdirected immune phenotype, which, when further compromised by heat stress, could exacerbate inflammation or reduce bacterial clearance, contributing to worsened outcomes. Additionally, heat stress may alter the immune microenvironment [46], potentially compromising lymphocyte efficacy and allowing MTB to persist or reactivate. In this context, higher lymphocyte levels may not protect the host as expected. Instead, they may change the immune response in a way that makes the body more sensitive to the harmful effects of temperature on the immune system. Future research should further investigate how environmental factors affect mycobacterial diseases across distinct immune phenotypes to determine which profiles are more susceptible to environmental stressors.

This research investigated the effects of environmental exposures and WBC profiles on pulmonary DS-TB, MDR-TB, and NTM, providing a foundation for future research. However, there are some limitations that must be acknowledged. The infection status of healthy controls was not determined; therefore, the control group may have included both uninfected and latently infected individuals. Consequently, our findings were interpreted as reflecting associations with clinically active mycobacterial disease, rather than progression from latent infection to active mycobacterial disease. Although environmental exposures were estimated using individual 30-day windows preceding diagnosis, which partially account for short-term variability, we acknowledge that residual seasonal confounding may remain since seasonal cycles were not explicitly modeled. Potential unmeasured confounders, such as comorbidities and socioeconomic status, could potentially affect occurrence of these diseases. Additionally, findings may not be fully generalizable across regions with varying environmental and healthcare conditions. Observational design also limits causal inference, requiring confirmation through longitudinal or randomized studies. Unlike vector-borne diseases with climate-driven seasonal patterns [47], TB is primarily socially influenced [48]. Future research should investigate the interplay between socio-environmental factors and TB.

## Conclusion

This study highlights how short-term environmental exposures interact with host immune responses, reflected by WBC, to influence pulmonary mycobacterial diseases, particularly noting that elevated neutrophil levels attenuate the impact of higher temperatures on NTM diagnosis, while increased lymphocyte levels amplify temperature-related associations with DS-TB and MDR-TB. These findings underscore the importance of integrating environmental and WBC data to inform TB prevention efforts and enhance public health strategies.

**Table 4:** Interaction effects of environmental factors with immune profiles on pulmonary TB, MDR-TB, and NTM

	DS-TB vs. Control OR (95%CI)	MDR-TB vs. Control OR (95%CI)	NTM vs. Control OR (95%CI)
Environmental factors*Neutrophils			
Humidity (%)	1.02 (0.99, 1.05)	1.00 (0.97, 1.04)	0.99 (0.95, 1.03)
Humidity difference (%)	1.02 (0.99, 1.04)	1.01 (0.98, 1.04)	1.02 (0.98, 1.05)
Temperature (°C)	0.97 (0.95, 0.99)	1.01 (0.99, 1.04)	0.96 (0.93, 0.99)
Temperature difference (°C)	0.95 (0.91, 0.99)	1.00 (0.95, 1.04)	**0.86 (0.81**, 0.91) *
PM_2.5_ (µg/m^3^)	1.01 (0.99, 1.03)	0.96 (0.94, 1.00)	0.99 (0.97, 1.02)
PM_2.5_ difference (µg/m^3^)	1.03 (1.00, 1.07)	1.01 (0.98, 1.03)	1.07 (1.00, 1.11)
Environmental factors*Lymphocytes			
Humidity (%)	1.02 (0.94, 1.11)	1.04 (0.94, 1.13)	0.94 (0.81, 1.08)
Humidity difference (%)	0.93 (0.87, 1.01)	1.08 (0.99, 1.17)	0.93 (0.82, 1.06)
Temperature (°C)	1.09 (1.02, 1.16)	1.08 (0.99, 1.18)	0.96 (0.84, 1.09)
Temperature difference (°C)	**1.25 (1.13**, 1.39) *	**1.17 (1.03**, 1.34) *	0.89 (0.74, 1.08)
PM_2.5_ (µg/m^3^)	0.92 (0.85, 1.00)	1.05 (0.96, 1.15)	0.98 (0.89, 1.07)
PM_2.5_ difference (µg/m^3^)	0.96 (0.88, 1.03)	**0.92 (0.87**, 0.98) *	0.95 (0.84, 1.07)
Environmental factors*Eosinophils			
Humidity (%)	1.03 (0.84, 1.24)	0.98 (0.80, 1.16)	1.15 (0.81, 1.65)
Humidity difference (%)	1.01 (0.86, 1.20)	1.06 (0.84, 1.32)	1.03 (0.71, 1.43)
Temperature (°C)	1.01 (0.82, 1.28)	1.06 (0.87, 1.34)	0.85 (0.56, 1.17)
Temperature difference (°C)	1.26 (0.86, 1.96)	1.17 (0.83, 1.89)	1.16 (0.66, 2.61)
PM_2.5_ (µg/m^3^)	1.08 (0.89, 1.35)	0.82 (0.60, 1.09)	0.94 (0.77, 1.32)
PM_2.5_ difference (µg/m^3^)	0.92 (0.76, 1.14)	0.85 (0.60, 1.12)	1.25 (0.93, 1.94)

Abbreviations: DS-TB, drug-susceptible tuberculosis; MDR-TB, multidrug-resistant tuberculosis; NTM, non-tuberculous mycobacteria; PM_2.5_, particulate matter of *<* 2.5 μm in aerodynamic diameter; CI, confidence interval; OR, Odds Ratio of the interaction (product) term between the environmental factors and white blood cell parameters in the regression model. Age, gender, BMI, and smoking status were adjusted for in the models. Bolded results are considered significant; * denotes adjusted *p* < 0.05 after Benjamini–Hochberg false discovery rate correction

## Supplementary Information

Below is the link to the electronic supplementary material.

Supplementary Material 1

## Data Availability

The datasets used and/or analyzed during the current study are available from the corresponding author on reasonable request.

## Abbreviations

TB: Tuberculosis
MTB: Mycobacterium tuberculosis
DS-TB: Drug-susceptible tuberculosis
MDR-TB: Multidrug-resistant TB
NTM: Non-tuberculous mycobacteria
RH: Relative humidity
PM_2.5_: Fine particulate matter of < 2.5 μm in aerodynamic diameter
WBCs: White blood cells
BMI: Body mass index
SD: Standard deviations
OR: Odds ratio
CI: Confidence interval
BH-FDR: Benjamini-Hochberg false discovery rate

## References

[1] Barberis I, Bragazzi NL, Galluzzo L, Martini M. The history of tuberculosis: from the first historical records to the isolation of koch’s Bacillus. J Prev Med Hyg. 2017;58(1):E9–12. 28515626 PMC5432783

[2] World Health Organization. WHO consolidated guidelines on tuberculosis. Module 4: treatment-drug-resistant tuberculosis treatment, 2022 update: World Health Organization. 2022 [Available from: https://www.who.int/publications/i/item/9789240063129 36630546

[3] Strollo SE, Adjemian J, Adjemian MK, Prevots DR. The burden of pulmonary nontuberculous mycobacterial disease in the united States. Ann Am Thorac Soc. 2015;12(10):1458–64. 10.1513/AnnalsATS.201503-173OC. 26214350 10.1513/AnnalsATS.201503-173OC PMC4627421

[4] Honda JR, Virdi R, Chan ED. Global environmental nontuberculous mycobacteria and their contemporaneous man-made and natural niches. Front Microbiol. 2018;9:2029. 30214436 10.3389/fmicb.2018.02029 PMC6125357

[5] Xiao Y, He L, Chen Y, Wang Q, Meng Q, Chang W, et al. The influence of meteorological factors on tuberculosis incidence in Southwest China from 2006 to 2015. Sci Rep. 2018;8(1):10053. 10.1038/s41598-018-28426-6. 29968800 10.1038/s41598-018-28426-6 PMC6030127

[6] Zhang CY, Zhang A. Climate and air pollution alter incidence of tuberculosis in Beijing, China. Ann Epidemiol. 2019;37:71–6. 10.1016/j.annepidem.2019.07.003. 31473123 10.1016/j.annepidem.2019.07.003

[7] Torres M, Carranza C, Sarkar S, Gonzalez Y, Osornio Vargas A, Black K, et al. Urban airborne particle exposure impairs human lung and blood Mycobacterium tuberculosis immunity. Thorax. 2019;74(7):675–83. 10.1136/thoraxjnl-2018-212529. 31036772 10.1136/thoraxjnl-2018-212529 PMC7162557

[8] Liu L, Xing X, Hu C, Wang H. One-year survey of opportunistic premise plumbing pathogens and free-living amoebae in the tap-water of one Northern City of China. J Environ Sci (China). 2019;77:20–31. 10.1016/j.jes.2018.04.020. 30573084 10.1016/j.jes.2018.04.020

[9] Lin K, Marr LC. Humidity-dependent decay of viruses, but not bacteria, in aerosols and droplets follows disinfection kinetics. Environ Sci Technol. 2019;54(2):1024–32. 10.1021/acs.est.9b04959 31886650

[10] Borkute RR, Woelke S, Pei G, Dorhoi A. Neutrophils in tuberculosis: cell biology, cellular networking and multitasking in host defense. Int J Mol Sci. 2021;22(9):4801. 10.3390/ijms22094801. 33946542 10.3390/ijms22094801 PMC8125784

[11] Bohrer AC, Castro E, Hu Z, Queiroz ATL, Tocheny CE, Assmann M, et al. Eosinophils are part of the granulocyte response in tuberculosis and promote host resistance in mice. J Exp Med. 2021;218(10):e20210469. 10.1084/jem.20210469. 34347010 10.1084/jem.20210469 PMC8348215

[12] Shu C-C, Wu M-F, Pan S-W, Wu T-S, Lai H-C, Lin M-C. Host immune response against environmental nontuberculous mycobacteria and the risk populations of nontuberculous mycobacterial lung disease. J Formos Med Assoc. 2020;119:S13–22. 32451216 10.1016/j.jfma.2020.05.001

[13] De Martino M, Lodi L, Galli L, Chiappini E. Immune response to Mycobacterium tuberculosis: a narrative review. Front Pead. 2019;7:350. 10.3389/fped.2019.00350 PMC6718705 31508399

[14] Zhang Y, Liu M, Wu SS, Jiang H, Zhang J, Wang S, et al. Spatial distribution of tuberculosis and its association with meteorological factors in Mainland China. BMC Infect Dis. 2019;19(1):379. 10.1186/s12879-019-4008-1. 31053104 10.1186/s12879-019-4008-1 PMC6500018

[15] Bonell A, Contamin L, Thai PQ, Thuy HTT, van Doorn HR, White R, et al. Does sunlight drive seasonality of TB in Vietnam? A retrospective environmental ecological study of tuberculosis seasonality in Vietnam from 2010 to 2015. BMC Infect Dis. 2020;20(1):184. 10.1186/s12879-020-4908-0. 32111195 10.1186/s12879-020-4908-0 PMC7048025

[16] Narasimhan P, Wood J, Macintyre CR, Mathai D. Risk factors for tuberculosis. Pulm Med. 2013;2013(1):828939. 10.1155/2013/828939. 23476764 10.1155/2013/828939 PMC3583136

[17] Cha J, Thwaites GE, Ashton PM. An evaluation of progress towards the 2035 WHO end TB targets in 40 high burden countries. medRxiv. 2020:2020.10.02.20175307. 10.1101/2020.10.02.20175307

[18] Thomson RM, Furuya-Kanamori L, Coffey C, Bell SC, Knibbs LD, Lau CL. Influence of climate variables on the rising incidence of nontuberculous mycobacterial (NTM) infections in Queensland, Australia 2001–2016. Sci Total Environ. 2020;740:139796. 32563864 10.1016/j.scitotenv.2020.139796

[19] Borgdorff MW, Sebek M, Geskus RB, Kremer K, Kalisvaart N, van Soolingen D. The incubation period distribution of tuberculosis estimated with a molecular epidemiological approach. Int J Epidemiol. 2011;40(4):964–70. 21441552 10.1093/ije/dyr058

[20] Makrufardi F, Chuang HC, Suk CW, Lin YC, Rusmawatiningtyas D, Murni IK, et al. Particulate matter deposition and its impact on tuberculosis severity: A cross-sectional study in Taipei. Sci Total Environ. 2024;924:171534. 10.1016/j.scitotenv.2024.171534. 38453064 10.1016/j.scitotenv.2024.171534

[21] Tsai CY, Su CL, Wang YH, Wu SM, Liu WT, Hsu WH, et al. Impact of lifetime air pollution exposure patterns on the risk of chronic disease. Environ Res. 2023;229:115957. 10.1016/j.envres.2023.115957. 37084949 10.1016/j.envres.2023.115957

[22] Griffith DE, Aksamit T, Brown-Elliott BA, Catanzaro A, Daley C, Gordin F, et al. An official ATS/IDSA statement: diagnosis, treatment, and prevention of nontuberculous mycobacterial diseases. Am J Respir Crit Care Med. 2007;175(4):367–416. 10.1164/rccm.200604-571ST. 17277290 10.1164/rccm.200604-571ST

[23] Migliori GB, Sotgiu G, Rosales-Klintz S, Centis R, D’Ambrosio L, Abubakar I, et al. ERS/ECDC statement: European union standards for tuberculosis care, 2017 update. Eur Respir J. 2018;51(5). 10.1183/13993003.02678-2017. 10.1183/13993003.02678-2017 29678945

[24] Siddiqi S, Ahmed A, Asif S, Behera D, Javaid M, Jani J, et al. Direct drug susceptibility testing of Mycobacterium tuberculosis for rapid detection of multidrug resistance using the Bactec MGIT 960 system: a multicenter study. J Clin Microbiol. 2012;50(2):435–40. 10.1128/JCM.05188-11. 22162558 10.1128/JCM.05188-11 PMC3264138

[25] Ho CC, Chen LJ, Hwang JS. Estimating ground-level PM(2.5) levels in Taiwan using data from air quality monitoring stations and high coverage of microsensors. Environ Pollut. 2020;264:114810. 10.1016/j.envpol.2020.114810. 32559863 10.1016/j.envpol.2020.114810

[26] Bai KJ, Liu WT, Lin YC, He Y, Lee YL, Wu D, et al. Ambient relative humidity-dependent obstructive sleep apnea severity in cold season: A case-control study. Sci Total Environ. 2023;861:160586. 10.1016/j.scitotenv.2022.160586. 36455744 10.1016/j.scitotenv.2022.160586

[27] Lin Y-C, Shih H-S, Lai C-Y, Tai J-K. Investigating a potential map of PM2. 5 air pollution and risk for tourist attractions in Hsinchu County, Taiwan. Int J Environ Res Public Health. 2020;17(22):8691. 33238515 10.3390/ijerph17228691 PMC7700626

[28] Wang XQ, Huang K, Cheng X, Hu CY, Ding K, Yang XJ, et al. Short-term effect of particulate air pollutant on the risk of tuberculosis outpatient visits: A multicity ecological study in Anhui, China. Atmos Environ. 2022;280:119129. 10.1016/j.atmosenv.2022.119129.

[29] Tran HM, Lin YC, Tsai FJ, Lee KY, Chang JH, Chung CL, et al. Short-term mediating effects of PM(2.5) on climate-associated COPD severity. Sci Total Environ. 2023;903:166523. 10.1016/j.scitotenv.2023.166523. 37625725 10.1016/j.scitotenv.2023.166523

[30] Tsai DH, Riediker M, Wuerzner G, Maillard M, Marques-Vidal P, Paccaud F, et al. Short-term increase in particulate matter blunts nocturnal blood pressure dipping and daytime urinary sodium excretion. Hypertension. 2012;60(4):1061–9. 10.1161/HYPERTENSIONAHA.112.195370. 22868388 10.1161/HYPERTENSIONAHA.112.195370

[31] Tran HM, Tsai F-J, Wang Y-H, Lee K-Y, Chang J-H, Chung C-L, et al. Joint effects of temperature and humidity with PM2. 5 on COPD. BMC Public Health. 2025;25(1):424. 39901163 10.1186/s12889-025-21564-3 PMC11789386

[32] Lam HC, Li AM, Chan EY, Goggins WB 3. The short-term association between asthma hospitalisations, ambient temperature, other meteorological factors and air pollutants in Hong kong: a time-series study. Thorax. 2016;71(12):1097–109. 10.1136/thoraxjnl-2015-208054. 27343213 10.1136/thoraxjnl-2015-208054

[33] Huang K, Yang XJ, Hu CY, Ding K, Jiang W, Hua XG, et al. Short-term effect of ambient temperature change on the risk of tuberculosis admissions: assessments of two exposure metrics. Environ Res. 2020;189:109900. 10.1016/j.envres.2020.109900. 32980000 10.1016/j.envres.2020.109900

[34] Togias AG, Naclerio RM, Proud D, Fish JE, Adkinson N, Kagey-Sobotka A, et al. Nasal challenge with cold, dry air results in release of inflammatory mediators. Possible mast cell involvement. J Clin Investig. 1985;76(4):1375–81. 2414318 10.1172/JCI112113 PMC424080

[35] Onozuka D, Hagihara A. Associations of day-to-day temperature change and diurnal temperature range with out-of-hospital cardiac arrest. Eur J Prev Cardiol. 2017;24(2):204–12. 27798364 10.1177/2047487316674818

[36] Pan R, Gao J, Wang X, Bai L, Wei Q, Yi W, et al. Impacts of exposure to humidex on the risk of childhood asthma hospitalizations in Hefei, china: effect modification by gender and age. Sci Total Environ. 2019;691:296–305. 31323575 10.1016/j.scitotenv.2019.07.026

[37] Li Z, Liu Q, Chen L, Zhou L, Qi W, Wang C, et al. Ambient air pollution contributed to pulmonary tuberculosis in China. Emerg Microbes Infect. 2024;13(1):2399275. 10.1080/22221751.2024.2399275. 39206812 10.1080/22221751.2024.2399275 PMC11378674

[38] Byber K, Radtke T, Norbäck D, Hitzke C, Imo D, Schwenkglenks M et al. Humidification of indoor air for preventing or reducing dryness symptoms or upper respiratory infections in educational settings and at the workplace. Cochrane Database Syst Reviews. 2021;(12). 10.1002/14651858.CD012219.pub2 PMC8664457 34891215

[39] Guarnieri G, Olivieri B, Senna G, Vianello A. Relative humidity and its impact on the immune system and infections. Int J Mol Sci. 2023;24(11):9456. 37298409 10.3390/ijms24119456 PMC10253274

[40] Salana S, Yu H, Dai Z, Subramanian PG, Puthussery JV, Wang Y, et al. Inter-continental variability in the relationship of oxidative potential and cytotoxicity with PM2. 5 mass. Nat Commun. 2024;15(1):5263. 38898130 10.1038/s41467-024-49649-4 PMC11187120

[41] Lin H, Ma W, Qiu H, Vaughn MG, Nelson EJ, Qian Z, et al. Is standard deviation of daily PM2. 5 concentration associated with respiratory mortality? Environ Pollut. 2016;216:208–14. 27262134 10.1016/j.envpol.2016.05.068

[42] Kroon EE, Coussens AK, Kinnear C, Orlova M, Möller M, Seeger A, et al. Neutrophils: innate effectors of TB resistance? Front Immunol. 2018;9:2637. 30487797 10.3389/fimmu.2018.02637 PMC6246713

[43] de Martino M, Lodi L, Galli L, Chiappini E. Immune response to Mycobacterium tuberculosis: A narrative review. Front Pediatr. 2019;7:350. 10.3389/fped.2019.00350. 31508399 10.3389/fped.2019.00350 PMC6718705

[44] Yoon N-B, Son C, Um S-J. Role of the neutrophil-lymphocyte count ratio in the differential diagnosis between pulmonary tuberculosis and bacterial community-acquired pneumonia. Annals Lab Med. 2013;33(2):105. 10.3343/alm.2013.33.2.105 PMC3589634 23482854

[45] Li F, Chen D, Zeng Q, Du Y. Possible mechanisms of lymphopenia in severe tuberculosis. Microorganisms. 2023;11(11):2640. 38004652 10.3390/microorganisms11112640 PMC10672989

[46] Cantet JM, Yu Z, Rius AG. Heat stress-mediated activation of immune-inflammatory pathways. Antibiot (Basel). 2021;10(11):1285. 10.3390/antibiotics10111285. 10.3390/antibiotics10111285 PMC8615052 34827223

[47] Li Y, Dou Q, Lu Y, Xiang H, Yu X, Liu S. Effects of ambient temperature and precipitation on the risk of dengue fever: A systematic review and updated meta-analysis. Environ Res. 2020;191:110043. 10.1016/j.envres.2020.110043. 32810500 10.1016/j.envres.2020.110043

[48] Abhimanyu, Coussens AK. The role of UV radiation and vitamin D in the seasonality and outcomes of infectious disease. Photochem Photobiol Sci. 2017;16(3):314–38. 10.1039/c6pp00355a. 28078341 10.1039/c6pp00355a

