# Supplementary material for: Interaction effects of environmental factors with white blood cell profiles on mycobacterial pulmonary diseases: a case-control study
Source: BMC Infect Dis. 2025 Dec 23;26:152. doi: 10.1186/s12879-025-12375-3 (PMC12836817; doi:10.1186/s12879-025-12375-3)
Supplement: Supplementary file 1 — Supplementary Material 1 [file 12879_2025_12375_MOESM1_ESM.docx]

Table S1. Association of environmental factors with pulmonary NTM after Propensity Score Matching (PSM)

|  | NTM vs Control  OR (95%CI) |
| --- | --- |
| Humidity (%)  Humidity difference (%)  Temperature (°C)  Temperature difference (°C)  PM_2.5_ (μg/m^3^)  PM_2.5_ difference (μg/m^3^) | 0.98 (0.88, 1.09)  1.07 (0.98, 1.18)  0.94 (0.86, 1.03)  **1.20 (1.04, 1.39) ***  **1.15 (1.07, 1.26) ***  1.05 (0.96, 1.15) |

Abbreviations: PM_2.5_, particulate matter of *<*2.5 μm in aerodynamic diameter; NTM, non-tuberculous mycobacteria; OR, Odds Ratio; CI, confidence interval. Age, gender, BMI, and smoking status were adjusted for in the models. Bolded results are considered significant; * denotes adjusted p < 0.05 after Benjamini–Hochberg false discovery rate correction.

Table S2. Association of white blood cell profile with pulmonary NTM after PSM

|  | NTM vs Control  OR (95%CI) |
| --- | --- |
| Neutrophils (10³ cells/μL)  Lymphocytes (10³ cells/μL)  Eosinophils (10³ cells/μL) | **1.58 (1.24, 2.12) ***  **0.37 (0.20, 0.64) ***  2.09 (0.38, 17.40) |

Abbreviations: NTM, non-tuberculous mycobacteria; CI, confidence interval; OR, Odds Ratio. Age, gender, BMI, and smoking status were adjusted for in the models. Bolded results are considered significant; * denotes adjusted p < 0.05 after Benjamini-Hochberg false discovery rate correction.

Table S3. Interaction effects of environmental factors and immune profiles on pulmonary NTM after PSM

|  | NTM vs Control  OR (95%CI) |
| --- | --- |
| Environmental factors*Neutrophils | |
| Humidity (%) | 0.94 (0.87, 1.01) |
| Humidity difference (%) | 0.99 (0.93, 1.06) |
| Temperature (°C) | 1.01 (0.96, 1.07) |
| Temperature difference (°C) | **0.86 (0.77, 0.95) *** |
| PM_2.5_ (μg/m^3^) | 0.98 (0.94, 1.03) |
| PM_2.5_ difference (μg/m^3^) | 1.04 (0.98, 1.12) |
| Environmental factors*Lymphocytes | |
| Humidity (%) | 0.91 (0.78, 1.05) |
| Humidity difference (%) | 0.90 (0.77, 1.05) |
| Temperature (°C) | 0.93 (0.80, 1.07) |
| Temperature difference (°C) | 0.90 (0.73, 1.11) |
| PM_2.5_ (μg/m^3^) | 0.97 (0.87, 1.07) |
| PM_2.5_ difference (μg/m^3^) | 0.98 (0.86, 1.11) |
| Environmental factors*Eosinophils | |
| Humidity (%) | 1.23 (0.68, 2.34) |
| Humidity difference (%) | 0.83 (0.52, 1.32) |
| Temperature (°C) | 0.77 (0.42, 1.35) |
| Temperature difference (°C) | 1.26 (0.64, 3.13) |
| PM_2.5_ (μg/m^3^) | 0.99 (0.76, 1.48) |
| PM_2.5_ difference (μg/m^3^) | 1.44 (0.91, 2.68) |

Abbreviations: NTM, non-tuberculous mycobacteria; PM_2.5_, particulate matter of *<*2.5 μm in aerodynamic diameter; CI, confidence interval; OR, Odds Ratio of the interaction (product) term between the environmental factors and white blood cell parameters in the regression model. Age, gender, BMI, and smoking status were adjusted for in the models. Bolded results are considered significant; * denotes adjusted p < 0.05 after Benjamini-Hochberg false discovery rate correction.

# Shuang Ho Hospital, Ministry of Health and Welfare - Tucheng Health Care Generation Questionnaire

(Questionnaire explanation)

Hello! In light of the increasing aging population and the growing demand for long-term care in the community, Shuang Ho Hospital, Ministry of Health and Welfare, will establish the "Tucheng Health Care Generation " program. This program will include health promotion activities and physical examinations. Through long-term generational follow-up, we aim to explore changes in the health status of residents in the Tucheng area and identify causally related risk factors as a reference for disease prevention. Your personal information and opinions will be kept strictly confidential. Please feel free to answer truthfully. Thank you for your support and cooperation!

**Part One: Basic Information**

Shuang Ho Hospital, Ministry of Health and Welfare

Respectfully,

| Research ID: _____ Name: ___________________________________ |
| --- |
| Date of birth: ___Date_____ month_____ year Gender: □ Male □ Female |
| ID Number: _____ Contact Number: _________________________________ |
| Mailing Address: Tucheng District, New Taipei City_____ Street_____ Lane_________  ______________Number___________________Building |
| 1. Education level: □ Elementary school (or below) □ Junior high school  □ High school □ Junior college / university □ Graduate school or above |
| 2. Marital Status: □ Single □ Married □ Divorced □ Widowed □ Other |
| 3. Current employment status: □ Employed (Continue filling in "4") and question 4.1) □ Retirement □Household Management |
| 4. Occupation Category: □ Military □ Civil Service □ Education □ Business □ Industry □ Other |
| 4.1 My current (pre-retirement) job was from_____ to_____ |
| 5. Residence history: □ Same as the above mailing address □ Have moved (Continue to 5.1) question) |
| 5.1 Last residence address:  ________(County) District_____ Road (street) ______ Lane _____ Number______ Building |

### Part Two: Body composition measurement

| Height: _____cm Weight: _____kg Neck circumference: _____ cm |
| --- |
| Waist circumference: _____ cm Hips: _____cm Calf circumference: _____ cm |
| Blood pressure: _____ mmHg (systolic)/ _____ (diastolic) mmHg Pulse: _____beats /min |

**Part Three: Lifestyle Habits**

1. Do you have a habit of exercising? □ Yes □ No

2. The number of times I exercised in the last three months: □ None at all (Please skip to question 7) question)

□ One month 1~3 Weekly ​ 1-2 Weekly ​ 3-5 Almost every day

3. The degree and type of exercise achieved in each session are as follows:

□ Light exercise: regular walking, golf, gentle gymnastics, yoga, tai chi

□Moderate-intensity aerobic exercise: such as jogging, cycling, swimming, aerobic dance, and Yuanji dance.

□ Intermittent aerobic exercise that causes rapid breathing and sweating: such as tennis, basketball, volleyball, and table tennis.

□ Aerobic exercises that cause rapid breathing and sweating: such as running a marathon and athletic competition training.

4. Duration of each exercise session:

□ 1-10 Minutes □ 11-20 Minutes □ 21-30 minutes □ more than 30 minutes

5. Each exercise session should result in an increased heart rate, shortness of breath, slight fatigue, and sweating.

□ I don't know □ Rarely □ Often □ Almost always

6. Do you have a smoking habit (have smoked more than 100 cigarettes in total)?

□ No, I have never smoked, so please answer from question 10.

□ No, I have already quit smoking, and started quitting at the age of_____.

□ is

7. At what age did you start smoking? _____ age

8. How many cigarettes do you smoke on average per day? _____ (cigarettes / day)

9. Are you frequently exposed to secondhand smoke in your work or living environment? □ Yes □ No

(Frequent exposure: more than three days a week, and the habit has lasted for more than six months)

10. Do you have a habit of drinking alcohol?

Beer: □Never drink it □ 1-2 times a month □Once a week □ 2-3 times a week □ Almost every day

Fruit-based alcoholic beverages (such as wine, rice wine, red liqueur, Shaoxing wine, etc.)

□ Never drink it □ 1-2 times a month □ Once a week □ 2-3 times a week □ Almost every day

Spirits: (e.g., sorghum liquor, whiskey, brandy, Daqu liquor, Wujiapi, etc.)

□ Never drink it □ 1-2 times a month □ Once a week □ 2-3 times a week □ Almost every day

11. Do you have a habit of drinking coffee?

□Never drinks it □ 1-2 times a week Cup □ 3-4 times per week Cup □ every 5-6 weeks I drink cups almost every day.

If so, at what age did you start drinking coffee?

12. Do you have a habit of chewing betel nuts?

□ No, I have never had the habit of chewing betel nut. Please refer to point 14. Start answering the question

□ No, I've already quit and started quitting at age of .

□ Yes

13.1 How many betel nuts do you eat on average each day? grain

13.2 How many years have you been chewing betel nuts? year

### Part Four: Dietary Habits

14.Are you a vegetarian? □ Yes □ No (Please start answering from question 15)

- 1. Vegetarian time: □Early morning vegetarian meal □First / Fifteenth day of the lunar month □Full vegetarian diet
  2. Vegetarian diet includes: □ Lacto- ovo vegetarian diet □ No eggs or dairy products

1. Do you frequently consume fermented soy products? (e.g. , fermented bean curd, stinky tofu, miso )

□ None □Rarely consumed □Frequently consumed (more than once a week)

1. Do you frequently consume pickled foods? (e.g., pickled vegetables, sausages, bacon, ham, etc.)

□ None □Rarely consumed □Frequently consumed (more than once a week)

1. Do you frequently consume foods containing artificial sweeteners (saccharin)? (For example, candied fruit)

□ None □Rarely consumed □Frequently consumed (more than once a week)

18. Do you frequently eat smoked or grilled foods? (e.g., smoked meat, grilled meat)

□ None □ Rarely eat □ Eat frequently (more than once a week)

19. Do you often eat salted fish?

□ None □ Rarely eat □ Eat frequently (more than once a week)

20. Do you often eat fried food?

□ None □Rarely consumed □Frequently consumed (more than once a week)

### Part 5: Personal Medical History

| 21. Have you ever been diagnosed with this condition by a doctor?  (If so, please continue with questions 21.1 to 21.4) | | | 21.1  How many years have it been since this illness was diagnosed? | 21.2  Have you seen a doctor for this illness in the past year or so? | | 21.3  Is there currently any regulation?  Should I take medication? | | 21.4  Do your parents or siblings have this disease? | | |
| --- | --- | --- | --- | --- | --- | --- | --- | --- | --- | --- |
| A. Hypertension | □ Yes | □ None |  | □ Yes | □ None | □ Yes | □ None | □ Yes | □ None | □ I don't know |
| B. Diabetes | □ Yes | □ None |  | □ Yes | □ None | □ Yes | □ None | □ Yes | □ None | □ I don't know |
| C. Gout / High uric acid | □ Yes | □ None |  | □ Yes | □ None | □ Yes | □ None | □ Yes | □ None | □ I don't know |
| D. Anemia | □ Yes | □ None |  | □ Yes | □ None | □ Yes | □ None | □ Yes | □ None | □ I don't know |
| E. High blood lipids (e.g., cholesterol, triglyceride) | □ Yes | □ None |  | □ Yes | □ None | □ Yes | □ None | □ Yes | □ None | □ I don't know |
| F. Cerebrovascular diseases (e.g., stroke) | □ Yes | □ None |  | □ Yes | □ None | □ Yes | □ None | □ Yes | □ None | □ I don't know |
| G. Ophthalmic diseases (e.g., retinopathy) | □ Yes | □ None |  | □ Yes | □ None | □ Yes | □ None | □ Yes | □ None | □ I don't know |
| H. Heart disease/cardiovascular diseases | □ Yes | □ None |  | □ Yes | □ None | □ Yes | □ None | □ Yes | □ None | □ I don't know |
| I. Lungs (e.g., chronic bronchitis, asthma or chronic obstructive pulmonary disease) | □ Yes | □ None |  | □ Yes | □ None | □ Yes | □ None | □ Yes | □ None | □ I don't know |
| J. Liver disease | | | | | | | | | | |
|  Hepatitis B | □ Yes | □ None |  | □ Yes | □ None | □ Yes | □ None | □ Yes | □ None | □ I don't know |
|  Hepatitis C | □ Yes | □ None |  | □ Yes | □ None | □ Yes | □ None | □ Yes | □ None | □ I don't know |
|  Cirrhosis | □ Yes | □ None |  | □ Yes | □ None | □ Yes | □ None | □ Yes | □ None | □ I don't know |
| K. Kidney disease (e.g., glomerulonephritis, proteinuria, hematuria, polycystic disease) | □ Yes | □ None |  | □ Yes | □ None | □ Yes | □ None | □ Yes | □ None | □ I don't know |
| L. Urinary system (e.g., stones, infection) | □ Yes | □ None |  | □ Yes | □ None | □ Yes | □ None | □ Yes | □ None | □ I don't know |
| M. Prostate gland (e.g., hypertrophy, calcification) | □ Yes | □ None |  | □ Yes | □ None | □ Yes | □ None | □ Yes | □ None | □ I don't know |
| N. Bone and joint diseases | □ Yes | □ None |  | □ Yes | □ None | □ Yes | □ None | □ Yes | □ None | □ I don't know |
| O. Peripheral neuropathy | □ Yes | □ None |  | □ Yes | □ None | □ Yes | □ None | □ Yes | □ None | □ I don't know |
| P. Recurrent infections (e.g., skin, upper respiratory tract infections, etc.) | □ Yes | □ None |  | □ Yes | □ None | □ Yes | □ None | □ Yes | □ None | □ I don't know |
| Q. Autoimmune diseases (e.g.,lupus erythematosus) | □ Yes | □ None |  | □ Yes | □ None | □ Yes | □ None | □ Yes | □ None | □ I don't know |

**Part Six: Community Long-Term Care Services and Current Needs Survey**

Object: (1) 65 Seniors aged 60 or above or (2) those with experience using long-term care services

You or your family members used / received in the past 12 months?

□ I've never used it (skip to question 25) □ Yes

Who primarily uses the service: □ Yourself □ Family members

Items to be used: (Multiple choices)

□ (1) Home services □ (2) Day care services □ (3) Family care □ (4) Nutritional Catering Services

□ (5) Transportation pick-up and drop-off service □ (6) Purchase and rental of assistive devices and improvement of home accessibility

□ (7) Home-based integrated medical care □ (8) Home/ community rehabilitation □ (9) Respite care □ (10) Dementia care services

□ (11) Family caregiver support services □ (12) Other (please specify):

Regardless of whether you have used them before, which of the following items do you think are helpful for your or your family's care, and which would you consider using in the future? (Multiple selections allowed)

□ (1) Home services □ (2) Day care services □ (3) Family care □ (4) Nutritional Catering Services □ (5) Transportation pick-up and drop-off service □ (6) Purchase and rental of assistive devices and improvement of home accessibility □ (7) Home-based integrated medical care □ (8) Home/Community Rehabilitation □ (9) Respite care □ (10) Dementia care services □ (11) Family caregiver support services □ (12) Other ( please specify):

**@ Long-term care service description form**

| Services | Brief description |
| --- | --- |
| (1) Home services | A personal shopper provides in-home assistance with physical care and household chores. Physical care includes assisting with toileting, bathing, dressing, and eating; household chores include laundry, cleaning living areas, meal preparation, companionship, and grocery shopping.  Services such as providing necessities, accompanying patients to medical appointments, or contacting medical institutions. |
| (2) Day care services | This refers to the use of shuttle buses to transport elderly people with dementia or disabilities to day care centers during the day to receive specialized care.  This care service model includes services and health promotion, with family members or a shuttle bus picking up and dropping off the elderly at home in the evening. |
| (3) Family care | This refers to caregivers providing physical care, daily living assistance, and safety care to disabled elderly individuals within their residences and assisting them in participating in community activities according to their wishes and abilities. Services include physical care, daily living assistance, and safety care.  Daily living care and safety care: such as monitoring for abnormal situations, and urgently notifying medical institutions, etc. |
| (4) Nutritional catering services | It provides catering services for elderly people living alone or with disabilities, with options for group meals or home delivery. |
| (5) Transportation pick-up and drop-off service | Assisting moderately and severely disabled elderly people to access various long-term care services through transportation buses. |
| (6) Purchase, rental and home use of assistive devices and improvement of barrier-free environment | Subsidize the elderly to purchase or rent assistive devices and improve the barrier-free environment at home. |
| (7) Home-based integrated medical care | Based on public demand, various medical professionals, including physicians, home care workers, physical therapists, and pharmacists, etc., will provide services.  The case involves providing services and health education. |
| (8) Home / Community Rehabilitation | For disabled elderly individuals who are unable to go to the hospital for rehabilitation, professional physical therapists or occupational therapists will visit their homes or [other facilities].  The community provides rehabilitation training such as physical therapy and occupational therapy for people with disabilities. |
| (9) Respite services | The service aims to alleviate the caregiving burden on primary caregivers of disabled individuals. It is divided into two types: home-based and institutional respite. Home-based respite care involves caregivers providing services at the disabled individual's home, allowing the primary caregiver to rest. Institutional respite care involves temporarily placing the disabled individual in an institution for short-term or temporary care, allowing the primary caregiver to rest.  Those who are hired can have a period of rest. |
| (10) Dementia care services | It provides a diverse range of services for people with dementia, including home care, day care, family care, and small-scale multifunctional care.  In addition to group homes and other services, it reduces the burden of care for families. |
| (11) Family caregiver support services | The service targets family caregivers of disabled and demented elderly individuals, providing them with information on long-term care resources and services, including respite care, home care, and financial assistance. Professional staff will also be available to guide less experienced individuals in caregiving techniques.  They also offer various stress-relief activities and psychological counseling courses to provide emotional support to caregivers. |

##### Mini-Mental Health Inventory State Examination; MMSE​

The Mini-Mental State Assessment Scale includes items on orientation, attention, memory, language, oral comprehension, behavioral abilities, and constructive thinking. There is no time limit for the assessment, and the maximum score is 30. The score is 24 points, with higher scores indicating better cognitive function. One point is awarded for each correct answer, and a total score below 24 points is considered low. A score below 16 indicates that the individual has mild cognitive impairment; a score below 16 indicates mild cognitive impairment. A score of 1 indicates severe cognitive impairment.

| Evaluation Project | Assessment content | Score |
| --- | --- | --- |
| Orientation  (10) | 1. Time (5): "Can you tell me today's date?" Ask for any missing parts: year (1), month (1), day (1), week (1), season (1).  2 Location (5): "Where are you?" The omitted parts of the question: Province (1), City (1), Town (1), Hospital (1), Building (1). |  |
| Attention  (8) | Message registration (3): Clearly and slowly say the names of three unrelated objects, and then ask the client to repeat them once, for example: apple (1), watch (1), pen (1).  Series of minus 7 (5): Please have the client perform a series of minus 7 for a total of 5 times. One point is awarded for each correct answer (e.g., 100 – 7 – 7 – 7 – 7 – 7). Alternatively, the patient can be asked to read the numbers forwards or backwards.  "Harmony in the family brings prosperity in all things" or 5 A series of non-consecutive numbers. |  |
| Memory  (3) | Please repeat the names of the three items mentioned earlier, for example: apple (1), watch (1), pen (1). |  |
| Language  (5) | Naming (2): Show the client a watch and ask him what the item is called (1). Repeat (1) with a ballpoint pen.  Repeat (1): Please repeat the following: "Money can make the devil turn the millstone”.  Understanding (1): Show the client a piece of paper with "Close your eyes" printed in large print, ask the client to read it aloud, and then do as instruct.  Sentence writing (1): Please have the individual write a sentence. |  |
| Oral comprehension and capacity for action  (3) | Give the client a blank piece of paper without a pattern and say, “Take the paper with your right hand (1), fold it in half (1), and then put it on the floor (or give it to me again) (1)”. |  |
| Constructive power  (1) | Please have each individual draw the following overlapping pentagons onto a piece of white paper.  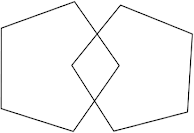 |  |
| Total Score | | |

References: Li Shih-Shih (2004) • Assessment of Long-Term Care Needs • Edited by Chen Hui-Tzu, Long-Term Care Practice (2nd Edition, 5-16~5-17) Page) ‧ Taipei: Yung -Da . Song Huijuan (2005) ‧ Assessment of long-term care for the elderly ‧ Yu Chen Qinghui et al., Long-term care (3rd edition, 350-351 Page) • Taichung: Wagner.

Note:

Maximum score 30 Points, 24-30​ A score of 12-24 is considered normal. A score of 9-12 is considered melancholic. The score is considered a possible sign of dementia requiring further evaluation; less than 9 This is considered a sign of dementia. Those with poor education levels are at least 15-17 years old. point.

(The numbers inside () represent the score for that item.

Message Login: After the test subject recites three words, ask them to remember them, explaining that they will be asked to recall these three words again in 3-5 minutes. If the test subject cannot completely recite the three words on the first attempt, they can repeat the practice up to 3 times, but the score will be based on the result of the first recitation.

7 " again during the calculation. Points are awarded based on whether the subtraction is correct each time. If the client needs to be reminded of the balance from the previous question, the current question will be incorrect. If both " 100-7 " and "subtract 7 " are incorrect, a different approach can be taken. The client can recite "Jiahe Wanshixing" and repeat the message.

Construction ability: Please draw the above figure on a piece of white paper (side length 3-5 cm). Then ask the test taker to draw the same figure as much as possible. Points will be awarded only if the two sides of the two pentagons intersect to form a quadrilateral. Points will be awarded regardless of size.

## Early Dementia Screening Scale (AD- 8)

**Screening date: Year Month Date**

1. Test Subject: □ The individual being tested

□ Caregiver or family member of the case (relationship to the case: )

##### 2. According to the Early Dementia Screening Scale (AD- 8) below, ask the patient (or caregiver or family member) whether there have been any changes in the following aspects in recent years. Record the results (1 point for changes, 0 points for no changes or not knowing.)

| Score | Topic | Content Description |
| --- | --- | --- |
|  | 1. Difficulty in judgment:  For example, falling into a trap or scam, making a bad financial decision, or buying something that is inappropriate for the recipient. | Compared to before, their "judgment" has deteriorated. For example, they are more easily scammed, make obviously wrong investments, or give a clock as a birthday gift, a dress as a gift to a boy, or an expensive gift to an unfamiliar friend. |
|  | 2. Interest in activities and hobbies decreases. | Compared to before, they seem less inclined to go out and have lost interest in activities they used to enjoy. However, this should be excluding factors such as changes in environment or limitations in mobility. For example, they used to frequently go to the activity center to sing karaoke, but now they don't want to go, and this isn't necessarily because of karaoke. |
|  | 3.Repeat the same questions, stories, and statements. | Compared to before, there are more instances of people repeatedly asking the same questions or recounting the same events. |
|  | 4.They have difficulty learning how to use tools, equipment, and small appliances. For example: television, air conditioner, washing machine, water heater, microwave oven, stereo, remote control. | Compared to before, the ability to use devices has decreased; for example, frequently dialing wrong numbers or being unable to make calls, and not knowing how to use the remote control to turn on the TV. This change in ability to use devices requires that the patient could use them in the past but cannot now; and this is not due to physical problems.  Caused by, for example, hand pain. |
|  | 5.Forgetting the correct month and year. | Compared to before, I used to be able to say the correct year and month, or I might say the wrong age. |
|  | 6.Difficulty handling complex financial matters. For example: balancing personal or family income and expenses, income tax, and utility bills. | Compared to before, it's more difficult to handle complex financial activities. For example, in the past, I was responsible for filing income tax returns, paying utility bills, and credit card bills, but now it's common for bills to be missed or overpaid.  Situations where money is paid or underpaid. |
|  | 7. It's difficult to remember the time of an appointment. | Compared to before, I now forget the times I'm supposed to meet with others more often. |
|  | 8. It has sustained thinking and memory abilities. | In recent years, I have been experiencing more and more problems with thinking or memory, such as the above-mentioned situations occurring more or less every day. |

Result: □ Score ≥ 2point, further diagnosis required □ Score < 2 point, and it is recommended to conduct at least one dementia screening per year.

This scale is only for preliminary screening of dementia and has no diagnostic significance. Please take your elderly relatives to major hospitals for further examination as soon as possible for early detection and early treatment!

Shuang Ho Hospital, Ministry of Health and Welfare, cares about you.

# Daily Living Activities Functioning Scale (ADL)

Evaluation Date: Year Month Date ​ ​

國

Case Name: Gender: Date of Birth:

| Item | Fraction | Content | | | | |
| --- | --- | --- | --- | --- | --- | --- |
|  | 10 | □They should be able to eat within a reasonable timeframe (approximately one bite every ten seconds), use chopsticks to pick up food in front of them, and be able to put on and take off eating utensils independently if needed.  □They need help putting on and taking off assistive devices or can only eat with a spoon.  □It takes too long for them to feed themselves. | | | | |
| I. Eating | 5 |  |  |  |  |  |
|  | 0 |  |  |  |  |  |
| II. Movement between wheelchair and bed | 15  10  5  0 | □It can be done independently, including braking the wheelchair and removing the foot pedals.  □They may need slight assistance (e.g., gentle support to maintain balance) or verbal instructions.  □Can sit up in bed on her own but still needs help to move around.  □Need help to sit up or two people to move. | | | | |
| III. Personal Hygiene | 5 | □It can independently complete tasks such as washing its face, washing its hands, brushing its teeth, and combing its hair. | | | | |
|  | 0 | □Need help from others | | | | |
|  | 10 | □Those who can enter and exit the toilet independently will not get their clothes dirty and can use the toilet while dressed properly can clean the toilet themselves.  □Those who use a bedpan can put the bedpan away themselves but must rely on others to clean it.  □Need help from others. | | | | |
| IV. Going to the toilet | 5 |  |  |  |  |  |
|  | 0 |  |  |  |  |  |
| V. Taking a bath | 5 | □It can be done independently (whether it's a bath or a shower). | | | | |
|  | 0 | □Need help from others. | | | | |
|  | 15 | □With or without assistive devices, one can walk independently for more than 50 meters.  □They need slight support or verbal guidance to walk more than 50 meters.  □Although unable to walk, he can operate the wheelchair independently (including turning, entering doors, and approaching tables and bed edges ) and can push the wheelchair for more than 50 meters.  □Need help pushing his wheelchair. | | | | |
| VI. Walking on flat ground | 10  5 |  |  |  |  |  |
|  | 0 |  |  |  |  |  |
|  | 10 | □Can go up and down stairs independently. (Holding onto handrails or using a cane is permitted.) | | | | |
| VII. Go up and down stairs | 5 | □Need a little help or verbal guidance | | | | |
|  | 0 | □Unable to go up or down the stairs. | | | | |
|  | 10 | □They can put on and take off their clothes, shoes, and assistive devices by themselves. | | | | |
| VIII. Put on and take off clothes | 5 | □With the help of others, one can complete half of the action on their own. | | | | |
|  | 0 | □Need help from others. | | | | |
|  | 10 | □It will not cause incontinence, and you can use suppositories on your own. | | | | |
| 9. Bowel control | 5 | □Occasionally, incontinence occurs (no more than once a week) or assistance is needed when using suppositories. | | | | |
|  | 0 | □Need to be handled by someone else. | | | | |
|  | 10 | □Do not have urinary incontinence day or night and may be able to use and clean diapers and diapers independently.  □Occasionally, there may be urinary incontinence (no more than once a week) or urinary urgency (unable to wait for the potty or get to the toilet in time) or the need for someone to help with the urinal.  □Need help from others. | | | | |
| 10. Urinary control | 5 |  |  |  |  |  |
|  | 0 |  |  |  |  |  |
| Total Score |  | | Assessment Unit |  | evaluator |  |

0 20 60 90 100

Fully dependent, heavily dependent, moderately dependent, functionally independent

##### Instrumental Activities of Daily Living (IADL)

The Long-Term Care Service Case Assessment Scale (96.11.15) was used.

| B. Instrumental Activities of Daily Living (IADL) | IADL) (based on performance  of the most recent month) |  |
| --- | --- | --- |
| 1. Shopping [ □ Not Applicable (If " Not Applicable " is selected, this item will be counted as full marks)]  □ 3. Independently complete all shopping needs  □ 2. Independently purchase daily necessities  □ 1. I need someone to accompany me every time I go shopping.  □ 0. Never goes shopping in public. | | Those who select 1 or 0 will be listed as having a disability. |
| 2. Outdoor Activities [ □ Not Applicable (If " Not Applicable " is selected, this item will be counted as full marks) ]  □ 4. Able to drive or ride a bicycle independently  □ 3. Able to take public transportation independently  □ 2. Able to take a taxi by themselves but not to use public transportation.  □ 1. When accompanied, a taxi or public transportation may be used.  □ 0. Completely unable to leave the house | | Those who select 1 or 0 will be listed as having a disability. |
| 3. Food preparation [ □ Not applicable (If " Not applicable " is selected, this item will be considered as full marks)]  □ 3. Able to independently plan, cook, and set out a suitable meal.  □ 2. If all the ingredients are prepared, a proper meal can be cooked.  □ 1. Will reheat prepared food.  □ 0. Need someone else to cook and set out the food. | | Those who select 0. will be listed as disabled. |
| 4. Household chores maintenance [ □ Not applicable (If " Not applicable " is checked, this item will be considered as full marks)]  □ 4. Able to do relatively heavy housework or need occasional assistance with housework (such as moving sofas, mopping floors, washing doors).  □ 3. Able to do simple housework, such as washing dishes, making the bed, and folding blankets.  □ 2. Able to do housework, but not to achieve an acceptable level of cleanliness.  □ 1. All household chores require assistance from others.  □ 0. Completely incapable of doing housework | | Those who select 1 or 0 will be listed as having a disability. |
| 5. Washing clothes [ □] not applicable (For those who select " Not Applicable ", this item will be considered as full marks.)  □ 2. Wash all clothes yourself  □ 1. Only wash small items of clothing.  □ 0. Completely dependent on others | | Those who select 0. will be listed as disabled. |
| 6. Telephone usage ability [ □ Not applicable (If " Not applicable " is checked, this item will be counted as full marks)]  □ 3. Independent telephone use, including looking up numbers in the phone book and dialing.  □ 2. Only dial familiar phone numbers.  □ 1. Can only answer the phone, not make calls.  □ 0. Completely unable to use a telephone | | Those who select 1 or 0 will be listed as having a disability. |

| 7. Taking medication [ □ Not applicable (If " Not applicable " is selected, this item will be counted as full marks)]  □ 3. Able to take responsibility for using the correct medication at the correct time.  □ 2. Need reminders or a little assistance  □ 1. If the dosage of medication has been prepared in advance, you can take it yourself.  □ 0. Cannot take medication on their own | Those who select 1 or 0 will be listed as having a disability. |
| --- | --- |
| 8. Financial Management Ability [ □ Not Applicable (If " Not Applicable " is selected, this item will be considered as full marks]  □ 2. Able to handle finances independently  □ 1. Can handle daily purchases but require assistance with bank transactions or large-scale deals.  □ 0. Inability to handle money | Those who select 0. will be listed as disabled. |
| Individuals who require assistance with three or more of the following five activities are considered to have mild disability: shopping, going out, cooking, housework, and laundry .) | |

#### Work stress Questionnaire - 1/2 page

Please check the appropriate options below with the “🗸” boxes

|  | **Subject** | **1**  **(No)** | **2**  **(Seldom)** | **3**  **(Sometimes)** | **4**  **(Often)** | **5**  **(Always)** | **5** |
| --- | --- | --- | --- | --- | --- | --- | --- |
| 1 | I know exactly what this job expects from me |  |  |  |  |  |  |
| 2 | I can decide when to take a break while working |  |  |  |  |  |  |
| 3 | I was overwhelmed by the demands of different unit at work |  |  |  |  |  |  |
| 4 | I know how to get my work done right |  |  |  |  |  |  |
| 5 | I have been subjected to personal harassment at work with unkind words or actions |  |  |  |  |  |  |
| 6 | I have unreasonable deadlines for completing my work |  |  |  |  |  |  |
| 7 | If I encounter difficulties at work, my colleagues will help me |  |  |  |  |  |  |
| 8 | I get support and feedback from my work |  |  |  |  |  |  |
| 9 | I have to work very hard to get my job done |  |  |  |  |  |  |
| 10 | I can decide how quickly I can work |  |  |  |  |  |  |
| 11 | I am well aware of my duties and responsibility at work |  |  |  |  |  |  |
| 12 | I had so much work to do that I had to ignore some |  |  |  |  |  |  |
| 13 | I am clear about the goals of my work department |  |  |  |  |  |  |
| 14 | There are often disputes and frictions between colleagues |  |  |  |  |  |  |
| 15 | I can decide for myself how to get the job done |  |  |  |  |  |  |
| 16 | I cannot get enough rest at work |  |  |  |  |  |  |
| 17 | I understand the relationship between my work and the overall goals of the department |  |  |  |  |  |  |
| 18 | I feel stressed by having to work long hours |  |  |  |  |  |  |
| 19 | I can decide what I work on |  |  |  |  |  |  |
| 20 | I had to work at a very fast pace |  |  |  |  |  |  |
| 21 | I was bullied at work |  |  |  |  |  |  |
| 22 | I have unrealistic time pressure at work |  |  |  |  |  |  |

#### Work stress scale Stress Questionnaire - Page 2 of 2

Please check the appropriate options below with the “🗸” boxes

|  | Subject | 1  No | 2  Seldom | 3  Sometimes | 4  Often | 5  Always |
| --- | --- | --- | --- | --- | --- | --- |
| 23 | When I have a problem at work, my direct supervisor will assist me |  |  |  |  |  |
| 24 | My colleagues will give me the help and support when I need |  |  |  |  |  |
| 25 | I can decide how I work |  |  |  |  |  |
| 26 | When there is a change in work, I have an opportunity to consult with my supervisor |  |  |  |  |  |
| 27 | I get the respect I deserve among my colleagues at work |  |  |  |  |  |
| 28 | When there are changes in work, the workplace often consults employees |  |  |  |  |  |
| 29 | I can talk to my direct supervisor about things at work that make me angry or upset |  |  |  |  |  |
| 30 | I have flexible working hours |  |  |  |  |  |
| 31 | My colleagues are willing to listen to my work problems |  |  |  |  |  |
| 32 | When there are changes on work, I know exactly how those changes are actually executed |  |  |  |  |  |
| 33 | Despite the psychological burden of work, I still felt supported |  |  |  |  |  |
| 34 | Relationships at work are tense |  |  |  |  |  |
| 35 | My direct supervisor encouraged me |  |  |  |  |  |

#### Depression Scale of the Epidemiological Research Center

##### Center for Epidemiologic Studies Depression Scale (CESD)

The following questions are some feelings or behaviors you may have recently experienced. Please tell us how many times you had these feelings last week.

| Question Number | Past feelings or past actions | Rarely or never | Very rarely | Quite a lot | Most of time |
| --- | --- | --- | --- | --- | --- |
| 1 | I used to be bothered by things that wouldn't normally bother me. | 1 | 2 | 3 | 4 |
| 2 | I used to not want to eat and had a very poor appetite. | 1 | 2 | 3 | 4 |
| 3 | Even with the help of family or friends, I still felt unable to shake off my depression. | 1 | 2 | 3 | 4 |
| 4 | I used to feel that I was just as good as everyone else. | 1 | 2 | 3 | 4 |
| 5 | I used to have difficulty concentrating on what I was doing. | 1 | 2 | 3 | 4 |
| 6 | I was frustrated | 1 | 2 | 3 | 4 |
| 7 | I used to feel that everything I did was very strenuous. | 1 | 2 | 3 | 4 |
| 8 | I was once full of hope for the future. | 1 | 2 | 3 | 4 |
| 9 | I used to think my life was a failure. | 1 | 2 | 3 | 4 |
| 10 | I was afraid | 1 | 2 | 3 | 4 |
| 11 | I used to have trouble sleeping | 1 | 2 | 3 | 4 |
| 12 | I was very happy | 1 | 2 | 3 | 4 |
| 13 | I spoke less than usual. | 1 | 2 | 3 | 4 |
| 14 | I have felt lonely and isolated. | 1 | 2 | 3 | 4 |
| 15 | People were unfriendly to me. | 1 | 2 | 3 | 4 |
| 16 | I used to enjoy the comforts of life | 1 | 2 | 3 | 4 |
| 17 | I cried in waves. | 1 | 2 | 3 | 4 |
| 18 | I felt sad | 1 | 2 | 3 | 4 |
| 19 | I used to feel that others didn't like me. | 1 | 2 | 3 | 4 |
| 20 | I used to lack the motivation to do anything. | 1 | 2 | 3 | 4 |

##### Epworth Sleepiness Scale

**Epworth Sleepiness Scale (ESS)**

Epworth Sleepiness Scale Sleepiness Scale (ESS) To help you assess how sleepy you are during the day, please select the frequency of your drowsiness in the following 8 scenarios.

0 = Never

1 = Occasionally (less than half the time)

2 = Very likely (about half the time)

3 = Often (rarely able to remain conscious)

| Context | Frequency of dozing off (0-3) |
| --- | --- |
| When sitting and reading |  |
| While watching TV |  |
| When sitting still in certain public places (such as in a theater or during a meeting) |  |
| While driving for an hour straight as a passenger |  |
| When you can lie down and rest in the afternoon |  |
| When sitting and talking to others |  |
| During quiet contemplation after lunch ( without drinking alcohol) |  |
| When traffic stops for a few minutes in the car |  |

##### Total Score : Note:

Total score of 10 A score of 10 or higher indicates that you have daytime sleepiness. If you also snore, you may have [a condition].

If you have sleep apnea, please consult a pulmonologist, otolaryngologist, neurologist, or psychiatrist to arrange a sleep study.

##### Pittsburgh Sleep Quality Chart

**Pittsburgh Sleep Quality Index (PSQI)**

Please answer the following questions regarding your daily (mostly) sleep habits over the past month:

1. When did you usually go to bed over the past month? Hours__ minutes
2. How long has it usually taken you to fall asleep over the past month? ___(minutes)
3. What time have you usually wake up in the morning over the past month? ___:___
4. Over the past month, how many hours of sleep have you actually managed each night? __:__

Please select one appropriate answer for each of the following questions. Please answer all of them.

1. Over the past month, have you experienced any of the following sleep problems, approximately several times per week?

|  | Never | Less than 2 times | Around 2 times | | 3 times or more | |
| --- | --- | --- | --- | --- | --- | --- |
| （1）Unable to fall asleep within 30 minutes | □ | □ | □ | □ | |  |
| （2) Wake up in the middle of the night or early morning | □ | □ | □ | □ | |  |
| （3) You have to get up and go to the toilet | □ | □ | □ | □ | |  |
| （4）Feeling that breathing is not smooth | □ | □ | □ | □ | |  |
| （5）Lound snoring or coughing | □ | □ | □ | □ | |  |
| （6）It will feel cold | □ | □ | □ | □ | |  |
| （7) Feeling irritable | □ | □ | □ | □ | |  |
| （8）Have nightmare | □ | □ | □ | □ | |  |
| （9）Pain in the body | □ | □ | □ | □ | |  |
| （10）Other | □ | □ | □ | □ | |  |

|  |
| --- |
|  |
|  |
|  |
|  |
|  |
|  |
|  |
|  |
|  |

Please explain:

1. Overall, how do you feel about your sleep quality over the past month?

□ Very good □ Not bad □ A little bad □ Very bad

1. Over the past month, how many nights a week have you typically needed medication to help you sleep?

□ Not occurred □ Less than once □ Twice □ Three or more times

1. Have you experienced drowsiness and inability to stay awake while dining, driving, or socializing in the past month?

How many times a week?

□ Not occurred □ Less than once □ Twice □ Three or more times

1. Over the past month, you may have felt listless and unable to get things done.

□ No □ Somewhat □ Yes □ Very serious

1. Do you have a sleeping partner or roommate?

□ No sleeping partner or roommate □ Sleeping partner or roommate in a different bedroom

□ Sleeping partner/roommate but not in the same bed □ Sleeping partner or roommate in the same bed

If you have a sleeping partner or roommate, please ask them and continue answering; how many times a week have the following situations occurred in the past month?

| Never | Less than 2 times | About  2 times | Three times or more |
| --- | --- | --- | --- |
|  |  |  |  |

1. Loud snoring □ □ □ □
2. I experienced brief periods of paused

breathing while falling asleep □ □ □ □

1. Twitching or trembling of the legs (including feet)

occurs while falling asleep. □ □ □ □

1. Feeling confused or unable to distinguish between

people, time and place when waking up at night. □ □ □ □

1. Other restlessness and agitation during sleep. □ □ □ □

Please explain:

## Respiratory Symptom Assessment Questionnaire

**A1. The feeling of panting (AF1, AF2), Sensation (SQ):**

| Serial number | Classification | project | lowest | Score (Please circle) | Highest |
| --- | --- | --- | --- | --- | --- |
| 1 | AF1 | The feeling of discomfort in breathing | No | 0-1-2-3-4-5-6-7-8-9- 10 | Unbearable |
| 2 | SQ1 | Accompanied by chest tightness (Tight) | none | 0-1-2-3-4-5-6-7-8-9- 10 | Very frequent or very serious |
| 3 | SQ2 | It feels very heavy and requires a lot of effort. | none | 0-1-2-3-4-5-6-7-8-9- 10 | Very frequent or very serious |
| 4 | SQ3 | Unable to breathe deeply, unable to get enough air | none | 0-1-2-3-4-5-6-7-8-9- 10 | Very frequent or very serious |
| 5 | SQ4 | The feeling of suffocation | none | 0-1-2-3-4-5-6-7-8-9- 10 | Very frequent or very serious |
| 6 | SQ5 | Rapid, deep and fast breathing | none | 0-1-2-3-4-5-6-7-8-9- 10 | Very frequent or very serious |
| 7 | SfQ6 | rapid, shallow breathing | none | 0-1-2-3-4-5-6-7-8-9- 10 | Very frequent or very serious |
| 8 | AF2-1​ | Feeling anxious | No | 0-1-2-3-4-5-6-7-8-9- 10 | Unbearable |
| 9 | AF2-2​ | Feeling frustrated | No | 0-1-2-3-4-5-6-7-8-9- 10 | Unbearable |
| 10 | AF2-3​ | Feeling scared | No | 0-1-2-3-4-5-6-7-8-9- 10 | Unbearable |

A 2. Factors related to wheezing

| Serial number | Classification | project | Options |
| --- | --- | --- | --- |
| 1 | Onset | It happened suddenly (0), took several days (1), several months (2), and several years (3). | 0, 1, 2, 3 |
| 2 | Triggering factors | Irritants (cold air, perfume) (0), after eating (1) | 0, 1 |
| 3 | Deterioration time | In the middle of the night (0, after getting up (1), while working (2),  After getting off work (3), after lying down to sleep (4) | 0, 1, 2, 3, 4 |
| 4 | Relief factor | Calm down and rest (0), sit up (1, lie down (2) | 0, 1, 2 |
| 5 | Related symptoms | Weakness in hands and feet (5-1), edema in both feet (5-2) General fatigue (5-3), wheezing during breathing (5-4),  Nasal congestion (5-5), burning sensation in the chest, acid reflux (5-6) | 5-1 (None /Yes), 5-2 (None / Yes)  5-3 (No / Yes), 5-4 (No / Yes  5-5 (None / Yes), 5-6 (None / Yes) |

**A3. Typical symptoms:**

| Serial number | project | lowest | Score (Please circle) | Highest |
| --- | --- | --- | --- | --- |
| 1 | He has little phlegm and coughs easily when he talks; he doesn't cough when he's not talking or sleeping. | Never before | 0-1-2-3-4-5​ | Always |
| 2 | I have a lot of phlegm, and it's darkest in color when I wake up in the morning. Sometimes I get short of breath when I walk. | Never before | 0-1-2-3-4-5​ | Always |
| 3 | Chest tightness or other discomfort during strenuous activities | Never before | 0-1-2-3-4-5​ | Always |
| 4 | After exercise, my breathing and coughing worsened, and I gradually found it difficult to breathe. I rested for more than ten minutes.  Improvement only later | Never before | 0-1-2-3-4-5​ | Always |
| 5 | I often feel short of breath and insufficient air intake when at rest;  However, there was no obvious difficulty breathing during exercise. | Never before | 0-1-2-3-4-5​ | Always |
| 6 | I cough as soon as I lie down to sleep, and I can only fall asleep after coughing up phlegm. | Never before | 0-1-2-3-4-5​ | Always |
| 7 | Sudden chest tightness and shortness of breath while sleeping, light sleep, vivid dreams, and noticeable snoring. | Never before | 0-1-2-3-4-5​ | Always |

**A4. Obstructive Pulmonary Questionnaire:**

| Serial number | project | (Please select) |
| --- | --- | --- |
| 1 | Have you ever lived or worked in an environment with poor air quality, smoking, secondhand smoke, or excessive dust? | (None / Yes) |
| 2 | Do your breathing symptoms change with the seasons, climate, or air quality? | (None / Yes) |
| 3 | Do your breathing symptoms prevent you from lifting heavy objects, shoveling dirt, jogging, playing tennis, or swimming? | (None / Yes) |
| 4 | Do you feel more tired than other people your age? | (None / Yes) |
| 5 | In the past 12 months, have you been unable to work or engage in normal activities several times due to colds, bronchitis, or pneumonia? | (None / Yes) |

## Environmental Fine Particulate Exposure Assessment Questionnaire

##### A1. Within a 50- meter radius of your current residence (within a 1- minute walk) , are there any of the following situations?

(Multiple Choice)

□ (01) Busy roads elevated roads or highways

□ (02) Restaurants or street vendors that produce cooking fumes

□ (03) Temples where people burn incense and pray

□ (04) Fixed night market or mobile night market (more than ten vendors or food and beverage establishments) at least once a week

□ (05) Other shops or factories that produce unpleasant smells ( odors ) or dust ( e.g., laundromats, motorcycle repair shops, etc.)

□ (06) None of the above

##### A2. Do you smoke?

□ (01) Currently drawing lots, one day's worth, one year's worth.

□ (02) I used to smoke, one pack a day, smoked for a year, quit for a year.

□ (03) Never smoke

□ (04) Secondhand smoke, □ Family members often smoke at home, or someone smokes indoors at work; □ Family members often smoke near the house.

##### A3. How many times did you clean your house in the past week? (Cleaning done by yourself doesn't count.)

□ (01) Daily, □ (02) Every 2-3 days, □ (03) Once a week, □ (04) Not cleaned

A4. Are you the main person in charge of cooking at home? □ (01) Yes, I cook every__ ____meals a week. □ (02) is not

A5. Do you worship gods / ancestors in your family (meaning you burn incense and offer sacrifices)? □ (01) Yes □ (02) No

A6. The place for worshipping gods / ancestors, the window □ Fully open or open □ (02) Half open □ (03) Small Opening □ (04) Close

A7. How many times a week should we worship deities / ancestors? ( Times per week)

##### A8. How many times a month do I go to the temple to pray ?

A9. How many times have you used traditional mosquito coils at home this week?

□ (01) daily □ (02) Once every 2-3 days □ (03) Once a week □ (04) Not used

A10. Did you use an air purifier at home this week?

□ (01) Yes; there is ____a platform (02 ) no

A11. Did you use a vacuum cleaner at home this week? □ (01) Yes □ (02) no

##### A12. On weekdays (working days / school days), how long do you spend on average outdoors?

□ (01) Less than 3 hours □ (02) 4~6 hours □ (03) 6~9 hours □ (04) More than 10 hours

##### A13. How long do you typically spend outdoors on average on weekends / holidays?

□ (01) Less than 3 hours □ (02) 4~6 hours □ (03) 6~9 hours □ (04) More than 10 hours

##### A14. Would you like to have the research team come to your home for a free air quality test?

□ (01) Yes, the respondent's contact number ; □ (02) No

### Weakness Index - Modified Fried Frailty index

| 1. Weight loss: □ No □ Yes □ Cannot be assessed  of 3 kg or more, or a weight loss of more than 5 %, in the past year without intentional weight loss.  If the answer to the above question is yes, the original weight was [weight] kg, and after [number] months, the current weight is [weight] kg. | | |
| --- | --- | --- |
| Weight loss | Weight loss of more than 1 kg compared to 3 years ago  (First ask the client if their weight is about the same as it was a year ago or has it decreased? If it has decreased, then ask approximately how much it has decreased.)  How many kilograms? | □ Yes (1 point)  □ No (0 point) |
| Lower limb function | Unable to stand up from the chair 5 times.  (While sitting in a chair, stand up 5 times without using your hands. Alternatively, if the case involves using a cane or walking aid, hook directly onto the chair.)  " yes ") | □ Yes (1 point)  □ No (0 points) |
| Reduce energy | Have you been feeling listless or unmotivated lately? | □ Yes (1 point)  □ No (0 points) |
| 2. Laborious: □ No □ Yes □ Unable to assess  Based on your answers to the following two questions (both answers are 2 or 3), how many days in the previous week did you feel the following?  (1) I feel that everything I do takes a lot of effort: ( )  (2) I have no way of starting anything: ( )  0: Rare or none (< 1) (day); 1: a little (1–2 day); 2: moderate (3–4 days) 3: Most of the time | | |
| 3. Physical activity level: □ No weakness □ Weak □ Unable to assess  Using Taiwan IPAQ Activity Level Survey (Self-administered Short Questionnaire for Seniors): Weekly Calorie Consumption ( kcal)  Reference values: US men: < 383 kcal indicates weakness; US women: < 270 kcal indicates weakness. | | |
| 4. 5 meter working time: □ No weakness □ Weakness □ Cannot be assessed  The number of seconds required to walk 5 meters: _____seconds  Reference value: Definition of male weakness in the United States: Height ≤ 173cm ≥ 7 seconds, height > 173cm The definition of a woman's weakness in the US for ≥6 seconds: height ≤ 159cm ≥ 7 seconds, height > 159cm ≥ 6 seconds  Time required to walk 6 meters: Second | | |
| 5. Grip strength (kg): □ No weakness □ Weak □ Cannot be assessed  Grip strength was measured using a JAMAR hand grip dynamometer.  Right hand: [< > + < > + < > ÷ 3 = ( ) Left hand: [< > + < > + < >] ÷ 3 = ( ) Reference value: American definition of male infirmity: BMI ≦ 24 ≤ 29, BMI 24.1-26 ≤ 30, BMI 26.1-28 ≤30, BMI​ > 28 ≦ 32  American definition of frailty in women: BMI ≦ 23 ≤17, BMI 23.1-26 ≤ 17.3  BMI 26.1-29 ≤ 18​ BMI > 29 ≦ 21 | | |
| Determination of weakness  □ No weakness (non- frail): 0 items  □ Pre -frail tendencies: 1 of the above Item or 2 item  □ Weak (frail): ≥ 3 item | | |

**Family Function Assessment Form**

Home is the place where we grow up, and the relationships with family members take many forms. (Answer format)

Please rate your current relationship with your surroundings. Each question is worth only one point, which is usually 2 points, sometimes 1 point, and rarely 0 points.

☐1. I am satisfied that I can turn to my family for help when I encounter difficulties. (Adaptability)

☐2. I am satisfied with the way my family and I discuss things and share problems. (Partnership)

☐3. Me when I wish to pursue new activities or explore new directions. (Growth)

☐4. Their reactions to my emotions (such as anger, sadness, and love). (Affinity)

☐5. I am satisfied with how my family gets along with me. (Resolve)

☐Total Score

scoring method for each question above.

A score of 7 to 10 indicates that the family is functioning without barriers.

A score of 4 to 6 indicates moderate family dysfunction.

A score of 0 to 3 indicates severe family dysfunction.

Quoted from S. MILKSTEIN, G. (1978).

Note: If you have family issues that require our assistance, please have a preliminary assessment by a doctor at a psychiatric outpatient clinic before being referred to a psychiatric social worker.

The social worker's office cares about you!

### Community Cohesion Chart

Questionnaire section (all questions are "single choice". Please mark " □ " with a thumbnail if you agree with your actual opinion

| Community Cohesion 2.0 Version /2013.06.20 | Completely disagree | disagree | ordinary | agree | Completely agree |
| --- | --- | --- | --- | --- | --- |
| 1 I will actively participate in various activities organized within the community. | □ | □ | □ | □ | □ |
| 2 I am happy to encourage my family or neighbors to participate in community activities. | □ | □ | □ | □ | □ |
| 3 My family approves of my participation in community activities. | □ | □ | □ | □ | □ |
| 4 I am happy to participate in community volunteer service groups. | □ | □ | □ | □ | □ |
| 5 I believe that community residents must help and support each other. | □ | □ | □ | □ | □ |
| 6 I believe that community matters are my matters. | □ | □ | □ | □ | □ |
| 7 If someone needs help, the community will be willing to offer assistance. | □ | □ | □ | □ | □ |
| 8 I pay attention to all the changes in my community. | □ | □ | □ | □ | □ |
| 9 I feel that my neighbors in the community are very kind to me. | □ | □ | □ | □ | □ |
| 10 I feel like I belong to the community I live in. | □ | □ | □ | □ | □ |
| 11 I can recognize most of the residents in the community. | □ | □ | □ | □ | □ |
| 12 I can easily find the exact location of every household in the community. | □ | □ | □ | □ | □ |
| 13 I believe my community is a great place to live. | □ | □ | □ | □ | □ |
| 14 I can clearly tell others how to get to my community. | □ | □ | □ | □ | □ |
| 15 When I'm in trouble, my neighbors lend a helping hand. | □ | □ | □ | □ | □ |
| 16 I often stop to chat with my neighbors. | □ | □ | □ | □ | □ |
| 17 My neighbors and I help each other. | □ | □ | □ | □ | □ |
| 18 When disputes arise between neighbors, I will take the initiative to help resolve them. | □ | □ | □ | □ | □ |
